# Tracking hemopexin intracellularly and defining hemopexin protein “interactomes” in human immune and liver cell models

**DOI:** 10.3389/fphys.2025.1613917

**Published:** 2025-11-20

**Authors:** Bryan Rose, David Moore, Jeff Eskew, Roberto Vanacore, Steve D. Hartson, Dennis Province, D. Andrew Skaff, Ann Smith

**Affiliations:** 1 UMKC School of Science and Engineering, Biological and Biomedical Systems, Kansas City, MO, United States; 2 UMKC School of Dentistry, Kansas City, MO, United States; 3 Poseida Therapeutics Inc., San Diego, CA, United States; 4 Division of Nephrology and Hypertension, Vanderbilt University Medical Center, Nashville, TN, United States; 5 Oklahoma State University, Stillwater, OK, United States; 6 IDeA National Resource for Quantitative Proteomics, U. of Arkansas for Medical Sciences, Little Rock, AR, United States; 7 Eir Pharmaceuticals, LLC, Olathe, KS, United States

**Keywords:** hemopexin, receptor, transferrin receptor 1, hemostasis, hemolysis, human hepatocytes, inflammation control, low density lipoprotein receptor 1

## Abstract

Maintaining hemopexin (HPX) plasma levels protects against heme-activated inflammation as well as the toxicity of heme and its iron during hemolysis. Plasma heme regulates HPX turnover in hepatocytes, thus controlling plasma HPX concentration. Heme from heme–HPX is delivered to the liver, and apo-HPX recycles without degradation. The scavenger receptor, low-density lipoprotein-related protein 1 (LRP1), binds heme–HPX and targets it for lysosomal degradation. Nevertheless, heme–HPX endocytosis also occurs in mouse embryonic fibroblast LRP1^−/−^PEA 13 cells. Therefore, the cell biology of heme–HPX endocytosis requires elucidation. We have identified candidate HPX receptors and human proteins that bind to heme–HPX, i.e., HPX “interactomes,” in a human neutrophil model (promyelocytic HL-60 cells), in hepatoma HepG2 cells, and in primary human hepatocytes. Immunoblots revealed that HL-60 cells lack LRP1, and immunocytochemistry established that HPX trafficked with transferrin and transferrin receptor 1 (TfR1) in Rab5-positive early endosomes, supporting a clathrin-mediated endocytotic pathway used by TfR1s. TfR1 was isolated by heme–HPX affinity chromatography of HL-60 and HepG2 extracts, and similarly, LRP1 from HepG2 cells. These receptors and novel HPX interactome proteins were identified by their peptide sequences. TfR1 downregulation in HL-60 cells in response to holo-human transferrin (Tf) decreased surface binding and intracellular HPX, implicating TfRs in heme–HPX endocytosis. In LRP^+/+^ HepG2 cells, HPX trafficked in endosomes with LRP1 and TfR1, or with TfR1 alone. HPX co-localized with TfR2, supporting that TfR2 potentially provides liver targeting of heme–HPX *in vivo*. TfR1 and 2 could both account for apo-HPX recycling. Heme–HPX affinity isolates from primary human hepatocytes contain LRP1 and TfR1. This HPX “interactome” also included proteins associated with hemostasis, inflammation control, coagulation regulation, wound healing, iron transport, and body fluid regulation. The overlapping and distinct roles of TfR1, TfR2, and LRP1 with HPX are reviewed. TfR1 is a scavenger receptor like LRP1; nevertheless, specific hepatic HPX receptors may exist. Increasing knowledge of HPX biology will elucidate the causes that regulate plasma HPX, thus improving clinical and veterinary care. Interestingly, increased understanding of the hematological adaptations to weightlessness that lead to anemia, termed “space anemia,” in astronauts and space tourists may provide new insights into HPX’s role in maintaining iron homeostasis and red cell biology under microgravity conditions as well as upon recovery from space and other anemias.

## Graphical Abstract

**Figure:**
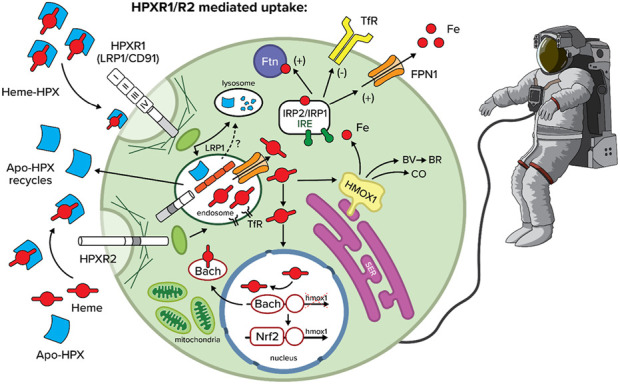
A new frontier for hemopexin? The extracellular binding of heme by hemopexin (HPX) and the delivery of heme to cells play key roles in the protective response of HPX during hemolysis and in the regulation of recovery from hemolysis. These functions of HPX are also important in minimizing the inflammation that may accompany red blood cell breakdown, trauma injury, and infection, irrespective of the cause. Two receptor-mediated uptake systems for heme–hemopexin (heme–HPX) endocytosis are depicted: via LRP1 (HPXR1) as the sole HPXR and HPXR2 for known apo-HPX recycling. In addition, some of the consequences are shown as the heme dissociates from HPX within maturing endosomes and is then broken down by heme oxygenase (HMOX1), with iron released. Intracellular routes of the receptors, heme–HPX, apo-HPX, and heme, derived from this research and published studies, are depicted. The HPX system may also contribute to a potential “lifeline” for astronauts and space tourists to overcome dysregulation of hemostasis by microgravity, including recovery from hemolysis and any resulting anemia.

## 1 Introduction

Both historical (Muller-Eberhard et al., 1968; Wochner et al., 1974) and recent research (Belcher et al., 2014) has established that it is the heme derived from hemoglobin that drives much of the cellular pathology in hemolytic diseases and conditions. The avid heme-binding protein, hemopexin (HPX, the apo-protein, *i.e.*, heme-free), present in plasma, cerebral spinal fluid, and lymph, protects cells in infection and injury by shielding against oxidative damage from infection and inflammation partly via immune cell activation by toll-like receptors (Belcher et al., 2014) not only in blood disorders but also in other conditions (Ajayi et al., 2025; Li et al., 2025; Zeng et al., 2025) and especially in critical illness (Lin et al., 2015) including trauma and sepsis (Larsen et al., 2010). Hemoglobin acts in different ways from heme to damage cells (Vallelian et al., 2022). HPX protection is especially important when the hemoglobin-binding protein, haptoglobin, is depleted (Miller et al., 1996).

Published research on the uptake of heme–HPX (*i.e.*, the 1:1 M ratio complex of heme and HPX) in a variety of human and animal cell types provides evidence for an interaction with one or more surface receptors. Heme–HPX causes biological effects, including signaling and gene regulation, in models of both human and rodent liver, neurons, and lymphocytes (Smith and Ledford, 1988; Smith et al., 1997; Eskew et al., 1999; Li et al., 2009), rat neuronal PC12 cells (Smith et al., 1997), and isolated rat hepatocytes (Liem, 1976; Li et al., 2009). HPX targeted heme to the liver in rats (Smith and Morgan, 1979), followed by recycling of intact apo-HPX; and in humans, HPX recycling has been implicated from plasma heme clearance studies (Drabkin, 1971). Transport protein recycling is analogous to, but not limited to, the well-characterized holo-transferrin (Tf)/transferrin receptor 1 (TfR1) system.

In cultured cells, heme–HPX binding and heme delivery initiate a “cytoprotective” program of events (Montecinos et al., 2019), including the induction of the heme degrading enzyme heme oxygenase (HMOX1). The iron from heme induces ferritin for iron storage (Davies et al., 1979) and downregulates transferrin receptors (TfRs), thereby minimizing iron uptake that might lead to iron overload. Iron may also increase ferroportin in liver cells for iron export and, consequently, the production of new red blood cells in the spleen and bone marrow. Furthermore, in response to heme–HPX endocytosis, there is copper-dependent induction of anti-oxidant metallothioneins (MTs, Alam and Smith, 1992), boosting protection against intracellular oxidative stress. Thus, heme–HPX endocytosis and heme delivery lead to the induction of several protective proteins (Larsen et al., 2010) and likely maintain intracellular levels of reduced glutathione.

Although the biochemistry of the HPX system has been studied in some detail, the cell biology of HPX has not been fully investigated. We have previously shown that heme–HPX complexes are co-localized with human Tf in coated pits, early endosomes, and multivesicular bodies (Smith and Hunt, 1990). These studies used electron microscopy with heme–HPX- or holo-human Tf-coated colloidal gold particles and, also, autoradiography of heme-^125^I-HPX in human hepatoma HepG2 cells. Overall, these data supported heme–HPX uptake by classical endocytosis and trafficking with Tf. However, HPX is targeted to lysosomes in Chinese hamster ovary (CHO) cells by the scavenger receptor, LRP1 (Hvidberg et al., 2005).

Heme–HPX-affinity chromatography was used to isolate putative HPX receptors (HPXRs) from pig liver (Majuri and Grasbeck, 1986), human placenta (Taketani et al., 1987a), and cell lines, including human promyelocytic HL-60 cells often referred to as myeloid leukemic cells (Taketani et al., 1987b). In most cases, an ∼80 kDa protein was found but was never identified. TfR1 is a homodimer of disulfide-linked 80 kDa subunits, and together with evidence for the recycling of apo-HPX and from immunocytochemistry (ICC), the findings implicate a role for TfR1 in the receptor-mediated endocytosis of heme–HPX. Nonetheless, the ubiquitous LRP1 was isolated by heme–human HPX affinity chromatography from CHO cells and shown by surface plasmon resonance to be a high-affinity HPX-binding protein, Kd 4 nM (Hvidberg et al., 2005). LRP1 consists of two non-disulfide-linked subunits of Mr 515 kDa and ∼85 kDa. It binds a plethora of more than a hundred ligands that vary widely in structure, and many are denatured proteins. In addition, LRP1 targets its ligands, including HPX, for degradation in lysosomes while LRP1 recycles to the cell surface (Hvidberg et al., 2005). However, we have shown that endocytosis of heme–HPX takes place in LRP1^−/−^ mouse fibroblast PEA13 cells (Smith, 2011). Thus, at least one other receptor system for heme-HPX uptake and apo-HPX recycling must exist.

With these observations in mind, we investigated the endocytosis and intracellular location of Alexa-Fluor (AF)-labeled heme–HPX with both Tf and TfR1 in LRP1^−/−^ HL-60 cells and LRP1^+/+^ HepG2 cells. Our data provide evidence of co-localization of Tf and TfR1 with HPX, supporting HPX’s transport along the same clathrin-mediated endocytotic pathway. Consistent with liver targeting, HPX was co-localized with TfR2 in HepG2 cells.

In addition, we carried out pilot studies to isolate heme–HPX-binding proteins (i.e., putative receptors and other biologically relevant HPX-interacting proteins—an “interactome”) using heme–HPX affinity chromatography of whole-cell extracts from three different human cell types: human promyelocytic HL-60 cells, human hepatoma HepG2 cells, and freshly isolated, plated human hepatocytes. Mass spectrometry (MS) and peptide sequencing of the isolated proteins identified TfR1 as the predominant novel receptor protein present in the HL-60 cell isolates from heme–HPX Affi-Gel 15, compared with those from several negative control Affi-Gels.

Overall, our combined ICC and MS data reveal that TfR1s were isolated in all three models of human cells. In the HepG2 and human liver affinity isolates, LRP1 was more abundant than TfR1, binding to either proto-heme-human or rabbit HPX complexes or complexes with mesoheme (Smith and Morgan, 1985). Specific interactions of heme–human HPX with proteins, *i.e.*, “the HPX interactome,” from HepG2 cells and primary human hepatocytes were investigated using ontology search line analysis, e.g., PANTHER GO Slim, with appropriate peptide coverage and significance (>100->90%). Proteins were grouped into biological roles, many consistent with the known functions of the HPX system but also including novel ones. We discuss the physiological and clinical relevance of these data and the potential future applications of these new findings on the HPX heme transport system from this pilot study that begins to identify human liver proteins involved in HPX biology.

## 2 Materials and methods

### 2.1 Proteins

Hemopexin: HPX was isolated from trace hemolyzed rabbit plasma (Pel-Freez; Rogers, AR) or human plasma (fresh frozen, Kansas City Blood Bank, KCMO). The formation and characterization of heme–HPX 1:1 complexes were carried out as described (Hahl et al., 2017). Proto-heme–human HPX, kindly provided by CSL Behring, Zurich, was used in certain experiments (Figures 3, 5).

Transferrin: to downregulate TfR1, cells were incubated overnight with 100 µM human holo-Tf (Sigma-Aldrich, United States) in endotoxin-free PBS (EMD Millipore) based on published studies. This holo-Tf was also used for the preparation of Affi-Gel 15 resins (see below).

### 2.2 Cell culture

Human promyelocytic HL-60 cells (Collins et al., 1977) and HepG2 cells, obtained from ATCC, were cultured as previously described (Alam and Smith, 1989). HepG2 cells were cultured in Eagle’s minimum essential medium (EMEM) supplemented with 10% FBS and 1% penicillin/streptomycin. Freshly isolated human hepatocytes from cooled, perfused human livers (obtained from BioIVT, Kansas City, KS, United States) were plated for culture in a defined serum-free Opti-Culture hepatocyte medium (BioIVT, Kansas City, KS, United States) in T-75 flasks or 6-well plates. For maximum recovery of liver functions (*e.g.*, cytochrome P450 system activity, BioIVT, Kansas City, KS, United States) in these cells, the defined medium was changed daily for 5 days before experimentation.

### 2.3 Initial affinity isolations of heme–HPX interacting proteins

**FIGURE 1:**
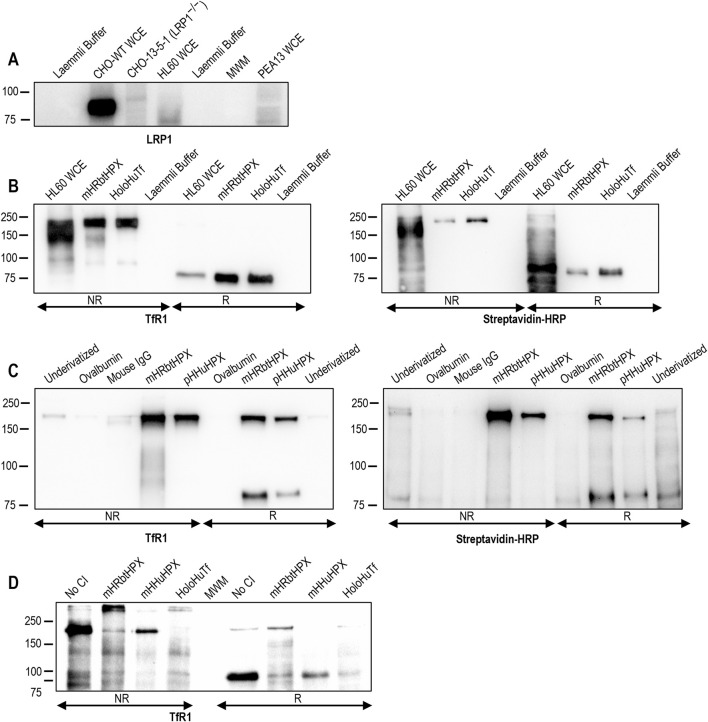
Affinity isolation of heme–HPX binding proteins from whole-cell extracts of LRP1^−/−^HL60 cells. **(A)** Western blotting reveals that HL-60 cells lack the small subunit of the scavenger receptor, LRP1, in contrast to wild-type (WT) CHO cells. Two additional LRP1 negative controls are the extracts of CHO-13-5-1 and human fibroblast PEA13 cells. **(B, C)** TfR1 is the protein bound to both mesoheme–rabbit HPX Affi-Gel (mHRbtHPX) and proto-heme–human HPX Affi-Gel 15 (pHHuHPX); furthermore, TfR1 is biotinylated, showing that it came from the cell surface of HL-60 cells (the concentration of the reducing agent was decreased to show both dimer and monomer of TfR1). Additional data are presented in Tables 1 and 2. **(D)** The binding specificity of the human TfR1 and heme–HPX interaction was confirmed by competitive inhibition of a mHRbtHPX Affi-Gel 15 with holoHuTf, as the positive control, or with both mHHuHPX and mHRbtHPX complexes. LRP1 (Mr ∼85 kDa), TfR1 homo-dimer (Mr ∼200–180 kDa), and TfR1 monomer (Mr ∼80 kDa).

Affi-Gel 15 resins were prepared following the manufacturer’s instructions (Bio-Rad, Hercules, CA, United States). First, gels with duplicate samples of mesoheme–rabbit HPX or ovalbumin affinity-isolated extracts from surface biotin-labeled HL-60 cells were run to determine the relative position of biotinylated proteins of interest by comparison with either unstained or pre-stained molecular weight protein standards (Bio-Rad). This allowed the determination of which gel regions contained protein HPXR targets compared with any proteins from the ovalbumin–Affi-Gel 15 samples residing in similar regions of the gel. To show the specificity of binding of proteins to the Affi-HPX ligand, we used competitive inhibition of resin binding of the cell biotinylated proteins by the addition of heme–rabbit–HPX, heme–human HPX, or holo-human Tf (final concentrations, ∼55–70 µM; Figure 1D; Supplementary Figure S1).

### 2.4 Preparation of whole-cell extracts for affinity isolations and analyses by immunoblotting

HL-60 whole cell extracts (500 µg protein) were incubated overnight at 4 °C with each set of protein Affi-Gel 15 beads (100 µL) in 50 mM HEPES/150 mM NaCl buffer, pH 7.4. After washing with ice cold HEPES/NaCl buffer, pH 7.4, bound proteins were eluted into Laemmle buffer (100 µL). For SDS-PAGE, the proteins in the Affi-eluates (10–20 µL) were reduced by β-mercaptoethanol or sodium dithionite (DTT, 100 mM), then separated by SDS-PAGE (8% gels), and transferred to PVDF membranes (0.2 micron). After incubation with HRP-labeled streptavidin (1:20,000 dilution; Thermo Fisher Scientific), the biotinylated proteins were detected by chemiluminescence (ECL, Sigma-Aldrich), following standard procedures using a Bio-Rad ChemiDoc MP scanner. Secondary antibodies (*e.g*., anti-rabbit IgG-HRP) were obtained from (Thermo Fisher Scientific, Lenxa, KS).

Heme–HPX complexes (30 mg, 15–20 mg/mL in PBS, pH 8.0) were coupled to 1.0 mL of washed Affi-Gel 15 beads (Bio-Rad), following the manufacturer’s instructions) for 1 h at 4 °C. After quenching with 100 µL of 1 M ethanolamine, the resin was then washed three times with HEPES-NaCl, pH 7.4. A similar procedure was used to produce the negative control resins: ovalbumin- and mouse IgG Affi-Gel 15 for the HPX receptor isolations, and for the positive control for TfR1, holo-human Tf Affi-Gel 15 (Sigma-Aldrich, St.Louis, MO) and mouse IgG from Thermo Fisher Scientific. Coupling efficiency was at least 65%–70%. The coupled resin was stored at 4 °C in buffered saline containing bactericidal agents, either thimerosal (heme-HPX-Affi-Gel) or sodium azide (negative controls). Protein concentrations were determined using the Pierce 660 nm protein assay (Pierce).

### 2.5 Affinity isolation of HPX binding proteins

We used well-controlled affinity chromatography to isolate putative hemopexin receptors (HPXRs) from whole cell extracts (100,000 x g isolates in HEPES buffered pH 7.4 Triton X-100 0.5%, 150 mM NaCl containing phosphatase and protease inhibitors, Millipore Sigma) of non-hepatic human promyelocytic^−^ HL-60, HepG2 cells, and freshly plated primary human hepatocytes. We followed the Affi-Gel 15 protocol (Bio-Rad) for coupling heme–HPX, holo–human Tf, ovalbumin, mouse IgG to the Affi-Gel 15 resin. In brief, Affi-Gel ligands in 50 mM HEPES buffer–150 mM NaCl, pH 7.4 (∼18 mg/mL) were added to a slurry of resin and incubated for 4 h at 4 C. Addition of 1 M ethanolamine in HEPES/NaCl blocked unreacted sites. The resin was then washed (50 mM HEPES buffer, 150 mM NaCl, pH 7.4) and stored at 4 °C.

### 2.6 Immunoblotting of affinity isolated heme–HPX binding proteins from HL-60 cells

HL-60 cell whole-cell extracts were used to determine whether these cells expressed LRP1. Cells grown in T75 flasks were scraped into lysis buffer. After centrifugation (3,000 × g for 10 min), protein concentrations were determined, as previously described by Rish et al. (2007). For LRP1 protein detection in whole-cell extracts, immunoblotting was carried out with primary antibodies: R2629 (rabbit polyclonal anti-LRP1 IgG, generated with the human heavy chain of LRP1 as antigen (Kounnas et al., 1992a; Kounnas et al., 1992b); 1 μg/mL dilution) or 5A6 (mouse monoclonal anti-LRP1 IgG generated with the light chain of human LRP1 as antigen; 1 μg/mL dilution). Secondary antibodies were HRP-conjugated goat anti-rabbit IgG (1:5,000 dilution, Thermo Fisher Scientific).

The surface biotinylation is described in Supplementary Material. To confirm that human TfR1 is present in the Affi-Gel 15-isolated proteins, including human TfR1 as known from the initial MS analyses, immunoblotting was carried out using anti-TfR1 (Abcam, Cambridge, MA, ab-84036, 1 μg/mL) and an HRP-linked second antibody (1:5,000 polyclonal rabbit anti-IgG, 65–6120, Thermo Fisher Scientific) using whole-cell extracts (15–20 µg) on 8% or 4%–20% SDS-PAGE gels, followed by transfer to PVDF membranes. Western analyses of both non-reduced and reduced samples were used to distinguish possible receptor subunits in whole-cell extracts of surface-biotinylated HL-60 cells. These were detected with streptavidin-HRP by chemiluminescence using a Bio-Rad ChemiDoc MP scanner (Bio-Rad). Pre-stained molecular weight standards were used (250 kDa–25 kDa range), and unstained protein markers (200 kDa–25 kDa range, Millipore) were detected after blotting using Ponceau red staining (data not shown). After detection using anti-TfR1, the PVDF blot was stripped using the Yeung and Stanley procedure (Yeung and Stanley, 2009). After checking the protein signals on the Bio-Rad ChemiDoc MP scanner, the membrane was then re-probed by incubation with streptavidin–HRP to reveal the affinity-isolated biotinylated proteins. To compare these samples, the images were merged using gel imaging software, Image Lab 6.0.1 software (Bio-Rad), to align the protein markers (from colorimetric scans) for molecular weight alignments before excising the gel regions for MS analyses and peptide sequencing to identify the proteins they contained.

### 2.7 Immunocytochemistry: preparation of Alexa Fluor-labeled proteins, cell surface binding, endocytosis, and downregulation of TfRs

Heme–HPX in PBS was fluorescently labeled using Alexa Fluor (AF) 546 or 647, according to the manufacturer’s instructions (Invitrogen). AF 488 holo-human Tf was purchased (Calbiochem). For fluorescence microscopy studies, HL-60 cells and HepG2 cells (seeding densities 1 × 10^4^ cells/well and 8 × 10^4^ cells/well, respectively) were placed in serum-free medium to facilitate their adhesion to coverslips and cultured on lysine-coated 8-well chamber slides (IBIDI) following the procedure detailed by Mihara et al. (2015). Cells were fixed and permeabilized (4% formaldehyde and 0.1% Triton-X 100/PBS 5 min at room temperature), followed by blocking (PBS, 0.1% Tween 20, 1% BSA, and 10% normal goat serum for 1 h or overnight at room temperature). Cells were stained with primary and secondary antibodies. For antibody information, please refer to Supplementary information.

### 2.8 Cell surface binding, endocytosis, and downregulation of TfRs

**FIGURE 2:**
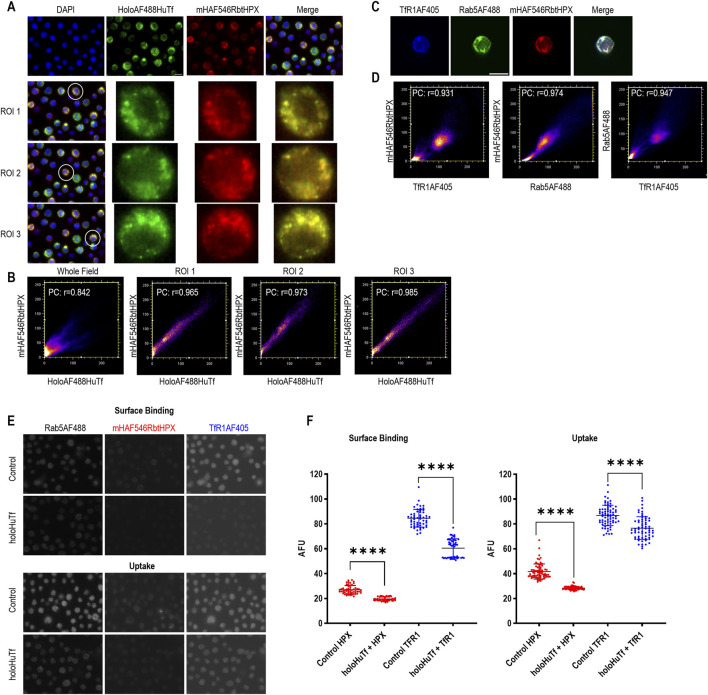
Evidence for trafficking of HPX with Tf and TfR1 support HPX internalization via the Tf endocytotic route in HL-60 cells. The data show co-localization within early Rab5-positive endosomes of HPX and Tf and HPX and TfR1. Significantly, the cell uptake of HPX is reduced after TfR1 downregulation in HL-60 cells, further supporting the involvement of TfRs in HPX endocytosis. **(A)** Co-localization of holoAF488HuTf (green color) with mHAF546RbtHPX (red color) after 10 min incubation at 37 °C. **(C)** Co-localization of TfR1AF405 (blue color) with mHAF546RbtHPX (red color) in early endosome marker Rab5AF488-positive (green color) endosomes. **(B, D)** Cytofluorograms of the whole field and regions of interest (ROIs) determine the overlay of Alexa Fluors and reveal positive Pearson coefficients (PCs). **(E, F)** Overnight incubation of HL-60 cells with 100 µM holo-human Tf significantly decreased TfR1AF405 (blue color) surface levels and uptake of mHAF546RbtHPX (red color). The early endosome marker Rab5AF488 was used as a mask to locate all the cells in the whole field. Thresholding methods were used to isolate pixels within these cells based on their higher intensity compared to background fluorescence. The results are presented as scatterplots, with error bars representing the mean ± SD of *n = 54* HL-60 cells from three separate fields for surface binding and *n = 74* HL-60 cells also from three separate fields for uptake, using Alexa Flour units (AFUs) on the y-axis. Significance is indicated as **** when *p* < 0.0001. Scale bar = 10 µm.

**FIGURE 3:**
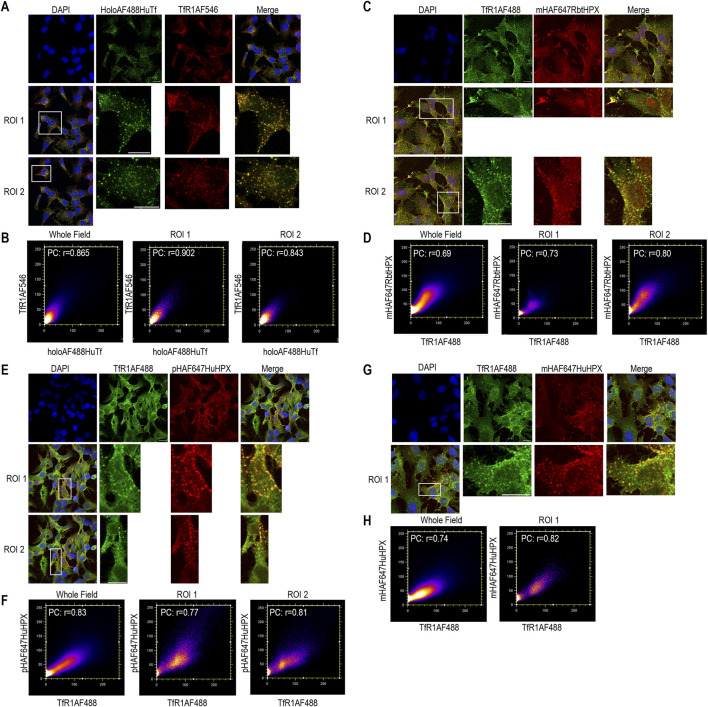
In HepG2 cells, ICC data support the rapid co-localization of TfR1 with human or rabbit HPX after uptake of their heme complexes as with the positive control ligand, holo-human transferrin. Cells were incubated with one of heme–HPX complexes for 10 min at 37 °C. TfR1 was identified using Alexa Fluor-labeled antibody as described in the Methods. **(A)** Co-localization holoAF488HuTf (green color) and TfR1AF546 (red color) after 10 min incubation at 37 °C as a positive control. **(C)** Co-localization of TfR1AF488 (green color) and mHAF647RbtHPX (red color) after 10 min incubation at 37 °C. **(E)** Co-localization of TfR1AF488 (green color) and pHAF647HuHPX (red color) after 10 min incubation at 37 °C. **(G)** Co-localization TfR1AF488 (green color) and mHAF647HuHPX (red color) after 10 min incubation at 37 °C. **(B, D, F, H)** Cytofluorograms of the whole field and ROIs that reveal a positive PC. HepG2 scale bar = 15 µm.

For surface binding and endocytosis of AF-labeled proteins, the cells were rinsed twice with ice-cold HEPES-buffered, serum-free media, pH 7.4, and incubated for 1 h on ice with either 500 nM heme–AF546HPX, heme–AF546HPX, or AF488 holo-human Tf. For endocytosis, cells were transferred to a 37 °C CO_2_ tissue culture incubator for 10 min, washed, and fixed. The cells were stained with primary and secondary antibodies. The hard set Vectashield mountant (Vector Labs) containing DAPI was used to stain nuclei. The HL-60 cells were visualized using a Nikon TE2000-U inverted microscope, while a Leica SP5 Laser Scanning Confocal Microscope was used for HepG2 cell experiments. For the ICC research with HepG2 cells, human and rabbit HPX isolated by the Smith group was used (500 nM), except for the ICC studies on binding and uptake in Figures 3, 5, where proto-heme–human HPX (CSL) was increased to 1 µM (Figure 3) or 2 µM (Figure 5) to increase the signal intensity. To downregulate TfRs and limit the amount of surface binding or endocytosis in HL-60 cells, 100 µM human holo-Tf (Sigma-Aldrich) in endotoxin-free PBS (EMD Millipore) was added to the medium for overnight incubation. Cells were washed twice with ice-cold HEPES-buffered serum-free media, pH 7.4, and either incubated for 1 h on ice with 500 nM heme–AF546HPX for surface binding or transferred to 37 °C for 10 min for endocytosis. To identify the intracellular localization of AF546HPX, HL-60 cells were co-stained with markers for early endosomes, *e.g.*, Rab5 antibodies (Jovic et al., 2010). To visualize the TfRs, cells were co-stained with AF-labeled antibodies to TfR1.

### 2.9 Contrast normalization and Gaussian Blur

Contrast normalization was used to assess the ICC images. The heme–human AFHPX complexes produced a fainter punctate signal on the HepG2 cells than the rabbit HPX complexes, necessitating the use of higher concentrations. We used thresholding for the quantification of TfR1 downregulation (Figure 2). The Rab5AF488 signal was used to set the mask for the Otsu threshold, which identified pixels that had sufficient signal and were considered parts of the cell, rather than pixels representing background values. Mean pixel values were used for data analysis. For a more detailed description of contrast normalization and Gaussian blur, see Supplementary Material and figure legends.

### 2.10 Statistical analyses of the ICC data

These analyses were carried out using GraphPad Prism. The Student’s unpaired *t*-test was used to determine significance, *e.g.*, the downregulation of TfR1 in HL-60 cells (*p*-values <0.05 were considered statistically significant). To further assess co-localization of target proteins in the ICC studies, we used ImageJ software; in addition, cytofluorograms provided an analysis of the extent of co-localization generating a Pearson coefficient. Additional information is provided in the figure legends.

### 2.11 Identification of affinity-isolated heme–HPX binding proteins by mass spectrometry

Three separate sets of adsorption reactions were used to obtain the initial HPXR isolates from HL-60 cells. The three separate pulldowns were assembled on different days, and the proteins were separated using SDS-PAGE. The indicated SDS-PAGE regions (see Supplementary Figure S1) were excised, reduced with tris (2-carboxyethyl) phosphine hydrochloride, alkylated with iodoacetamide, and digested with trypsin/LysC using standard methodologies. Peptides were separated on a 75 μm × 50 cm Acclaim PepMap C18 Column (Thermo Fisher Scientific) using a 4%–35% acetonitrile gradient for 120 min. During elution, peptides were ionized within a Nanospray Flex ion Source (Thermo Fisher Scientific) and analyzed by a “Top Speed” data-dependent MS/MS strategy, wherein precursor ions were measured within the Orbitrap sector at a nominal resolution of 120,000, concomitant with quadrupole selection, higher-energy collisional dissociation fragmentation, and fragment ion measurements within the ion trap sector. Peptides from each biological replicate were analyzed by three separate LC-MS/MS injections.

### 2.12 MS analyses of affinity-isolated proteins from HL-60 and HepG2 cells

After the initial protein analyses of the gel slices, washed Affi-resins from HL-60 cells and HepG2 cells were sent for MS analyses (Dr. S. Hartson). Additional Affi-resins from HepG2 cells were sent for analysis to CSL-Behring AG, Bern, Switzerland, and Affi-resins from primary human hepatocyte experiments were sent to Dr. D. Province (IDeA Proteomics). The original datasets from these analyses are available upon request to btrbkf@umkc.edu. Peptides from additional HepG2 cell extracts were first reduced with DTT, alkylated using iodoacetamide, and digested with trypsin using standard methodologies. Samples were analyzed using an Orbitrap Exploris 240 Mass Spectrometer (Thermo Fisher Scientific) connected to an Evosep One LC instrument (Evosep Biosystems). The standard “18SPD method” with an 88-min gradient was employed, utilizing a ReproSil-Pur C18 column (15 cm × 150 μm ID, 1.9 μm bead size) equipped with a 30 μm ID stainless steel emitter. The mass spectrometer operated in data-dependent acquisition (DDA) mode, with the following MS parameters: for MS1, the resolution was set to 120,000. The subsequent MS2 scan resolution was set to 30,000, acquiring as many scans as possible within a cycle time of 3s.

For HepG2 data searches, peptides were identified and quantified using MaxQuant (v1.5.10.3) to search the RAW instrument files against a database of 73,045 human proteins downloaded from Uniprot (Dr. S. Hartson). Data from each of the three bio-replicates were T-tested as one set (i.e., *p*-values validated the whole set of the three pulldowns as one big experiment (n1 = first pulldown, n2 = second pulldown, and n3 = third pulldown. The three technical replicates were categorized as individual experiments to support statistical testing of technical variance. Searches utilized the default MaxQuant settings, supplemented with biotinylation of lysine, acrylamide adducts of cysteine, and carboxymethylation of cysteine as additional variable modifications. Protein groups were identified and quantified as the simple sums of their peptide intensities without delayed normalization nor peptide ratio extraction (*i.e.*, data are the MaxQuant “protein intensities”). At CSF, injection replicates were used, and the search in MaxQuant included carbamidomethyl as a fixed modification and oxidation (M), acetyl (N-term) deamidation (N) and Gln- >PyroG-Glu as variable modifications. For additional information on sample treatment, please refer to Supplementary Material. Proteins that had a 2-fold or greater change (relative levels from the HPX Affi-isolates compared with the negative control isolates *e.g*., from Ovalbumin Affi-Gel 15) were considered specifically bound.

Please see Supplementary Material for further details of the MS analyses of samples from primary human hepatocytes.

### 2.13 Immunoblot detection of heme oxygenase 1

Heme-dependent heme-oxygenase 1 (HMOX1) induction was used to provide evidence of heme delivery by HPX to HepG2 and primary liver hepatocytes as described in the Supplementary Material.

### 2.14 Heparin affinity chromatography of HPX

Purified HPX (100 μL at 2 mg/mL) was chromatographed on a 1 mL HiTrap Heparin HP Column (GE Healthcare) at a flow rate of 0.5 mL/min on an AKTA Purifier 100 controlled with UNICORN 5.0 software. For additional experimental details, please refer to Supplementary Material. A known heparin binding protein, histidine proline-rich glycoprotein (HPRG), isolated from rabbit plasma, was used as a positive control (Lijnen et al., 1983; Borza and Morgan, 1998). To cover a range of biologically relevant pH, buffers used were 10 mM NaPO_4_ pH 7.4, 10 mM NaPO_4_ pH 6.6, and 10 mM MES pH 5.5. Apo- and mesoheme–rabbit HPX (∼90%) or proto-heme–human HPX complexes (33.9 mM and 38.3 mM, respectively) were investigated. The E_mm_ for HPRG was OD_280_ 0.53 = 1 mg/mL, and for hemopexin was OD_280_ 1.93 = 1 mg/mL. The protein elution profiles were recorded using OD_280_ for apo-HPX and for the heme complex, both OD_280_ and OD_404_. After sample protein injection, 2 column volumes (CV) of 10 mM sodium phosphate buffer, pH 7.4, were used to elute unbound material, followed by 10 CV of a gradient to 50% buffer containing 2 M NaCl (*i.e.*, a gradient from 0 M to 1 M NaCl). The initial conductivity of all buffers ranged from 0.25 to 1.9 mS/cm).

### 2.15 Competitive ligand binding studies of HPX with heparin

To assess the relative affinities of HPX for heparin and heme, aliquots of heparin were added to HPX in phosphate-buffered saline (4 °C), 45 min before mesoheme; the concentration of heme–HPX complexes was then determined by absorbance spectroscopy.

## 3 Results and discussion

The identification of surface receptors for heme–HPX complexes is of interest because HPX acts as an extracellular antioxidant against heme- and hemoglobin-mediated damage, including inflammatory states. This protective system is especially important when haptoglobin, which binds hemoglobin, is depleted or absent (Miller et al., 1996). The protective effects of HPX have been documented in a variety of clinical and experimental situations (Vinchi et al., 2016; Poillerat, 2020; Ashouri et al., 2021; Vallelian et al., 2022; Li et al., 2025), including cognitive impairment (Ashraf et al., 2020), and in helping maintain muscle quality (Zeng et al., 2025).

Clearly, HPX is linked to both the hepatic and immune systems. HPXRs play an important role in macrophage function in the immune system because heme from ^59^Fe–heme–HPX was efficiently incorporated into myeloperoxidase, a heme-protein in differentiated HL-60 cells (Vestal et al., 1990) and into cytochrome P-450 apo-protein (Correia et al., 2011). Myeloperoxidase produces hypohalous acid, an anti-microbial agent, and is stored in intracellular vesicles until it is released from neutrophils into mucus or sputum. This activity provides additional protection in pathological hemolytic conditions, sepsis, and possibly trauma.

### 3.1 Isolation and identification of heme–HPX binding proteins in human HL-60 cells

The HPX-binding scavenger receptor LRP1 is expressed by certain human immune cells, *e.g.*, neutrophil precursors, monocytes, and macrophages. Previously published binding experiments in HL-60 cells with heme-^125^I-HPX had revealed that these HPXRs (Taketani et al., 1987a; Taketani et al., 1987b) were of high affinity (∼1 nM) with *ca*. 40,000 receptors/cell. These are properties very similar to those reported for TfRs in HL-60 cells (Taetle et al., 1985), and apo-HPX is known to recycle after heme delivery to cells as does apo-Tf after iron delivery.

Because HPX is a highly conserved protein (Takahashi et al., 1985; Hahl et al., 2017) and its function to deliver heme to cells, especially hepatocytes, is maintained across species (Liem, 1976; Smith and Morgan, 1981), we used both proto- and meso-heme–rabbit (Rbt)HPX and human (Hu)HPX complexes that are biologically equivalent (Smith and Morgan, 1985). We first determined whether undifferentiated human promyelocytic HL-60 cells express LRP1 (Figure 1) using immunoblotting. In contrast to wild-type LRP1^+/+^CHO WT cells, HL-60 cells lack immunologically detectable LRP1, as do the LRP1^−/−^ CHO 13-51 cells engineered to lack LRP and the LRP1^−/−^ mouse fibroblast PEA13 cells (Figure 1A). Heme-Rbt and heme-HuHPX Affi-Gel 15 affinity-isolated proteins were fractionated using SDS-PAGE electrophoresis, transferred to PVDF membranes, and biotinylated proteins detected using streptavidin–HRP. Gel fractions corresponding to the biotinylated protein bands were excised (see Supplementary Figure S1), and the eluted proteins were analyzed by mass spectrometry Supplementary Table S1). To determine the gel regions to be excised for MS analyses, we used an overlay of the color image of pre-stained molecular weight (Mr) standards and the ECL signal (Supplementary Figure S1B). For better accuracy, we also used unstained proteins as molecular weight markers, detected after transfer to PVDF by Ponceau Red staining (not shown). From the apparent Mr of reduced and non-reduced HPXR isolates using the migration of unstained proteins markers, we anticipated that the novel HPXR is a disulfide-linked dimer of ∼90/100 kDa subunits, thus distinct from LRP1.

To identify the proteins present, database searches of the extracted proteins unambiguously identified the ∼200 kDa protein and ∼100 kDa proteins in non-reduced and reduced samples, respectively, as transferrin receptor 1 (TfR1, gene name TFRC), a ∼200-180 kDa homo-dimeric, disulfide-linked protein. Using SDS-PAGE analyses, the molecular weight of the human TfR1 dimer had been calculated to be 213 kDa and for the monomer 94 kDa (Enns and Sussman, 1981) to 95 kDa (Jing and Trowbridge, 1987). Significantly, TfR1 binding to the affinity column was contingent upon the presence of HPX (or holo-human Tf) and was not found in negative control eluates (*i.e.*, ovalbumin Affi-Gel 15 beads, see Supplementary Figure S1A, lanes 2 and 7).

To confirm the identify of TfR1 as a cell surface HPX-binding protein, blots of biotinylated HL-60 proteins were incubated with an anti-TfR1 antibody, then scanned and, after stripping (Yeung and Stanley, 2009), followed by streptavidin–HRP and rescanned. The signals produced by these separate detections revealed protein bands migrating close to ∼200 kDa and ∼90 kDa (non-reduced and reduced, respectively), as were proteins identified in eluates from holo-human transferrin (Tf) Affi-Gel 15, the positive control. The specificity of this interaction of heme–human HPX and of heme–rabbit HPX with TfR1 is shown by comparison with eluates from the negative control samples: ovalbumin Affi-Gel 15, mouse IgG Affi-Gel 15, and underivatized Affi-Gel 15 (Figures 1B,C). Competitive inhibition studies using either an excess of free heme–HPX ligands or with holo-human Tf prevented the binding of TfR1 (both monomer and dimer) to HPX, demonstrating the specificity of the interaction between these two proteins (Figure 1D). Overall, these data identify the biotinylated HPXR isolated from HL-60 whole-cell extracts using heme–HPX affinity chromatography as TfR1.

In addition, TfR1 was the most abundant protein in these ligand affinity isolates. Quantification and comparison of protein intensities from the ovalbumin pulldowns compared with the signal observed in the HPX affinity pulldowns showed that TfR1 recovery was highly specific and statistically significant (*p* = 0.0015, non-reducing, or *p* = 0.0027, reducing; and from Table 1, –log10 = 2.83 and 2.57, respectively). Importantly, several TfR1 peptides specific to the affinity pulldowns were identified as being biotin-labeled (see summaries of Table 1; Supplementary Table S1), showing that they were derived from the cell surface of LRP1^−/−^HL-60 cells. Additional interacting proteins not only confirm known functions of the HPX system but also reveal novel ones, some of which are cell- or tissue-specific. These data are presented in Section 3.2.

**TABLE 1:** Analysis and quantification by mass spectrometry of peptides identifying biotinylated transferrin receptor 1 (TfR1) isolated from the cell surface of biotinylated HL-60 cells from whole-cell extracts. Peptide identification scores and metrics were identified from the MaxQuant “Peptide” text output file. The complete dataset of biotinylated peptides from which these are derived is shown in Supplementary Table S1. Peptides were assigned to TfR1 comes based on localization probability, which is generally considered “good” at a value of ∼0.75 or higher.

PEP	Score	Score for localization	Localization probability	Intensity	Position	Protein	Protein name	Gene name
6.69E-10	204	203	1.00	4.10E+07	261	P02786	Transferrin receptor protein 1	TFRC
7.15E-04	143	102	1.00	1.22E+07	95	P02786	Transferrin receptor protein 1	TFRC
1.51E-04	140	134	0.99	7.17E+06	240	P02786	Transferrin receptor protein 1	TFRC
2.89E-08	195	195	1.00	5.05E+06	371	P02786	Transferrin receptor protein 1	TFRC
1.70E-26	220	150	1.00	4.60E+06	665	P02786	Transferrin receptor protein 1	TFRC
8.03E-03	145	120	1.00	2.71E+06	585	P02786	Transferrin receptor protein 1	TFRC
1.01E-02	100	89	0.62	6.01E+05	241	P02786	Transferrin receptor protein 1	TFRC

Receptor dimerization is often associated with signaling; therefore, because both heme–HPX or cobalt–protoporphyrin–HPX activate signaling cascades (Smith et al., 1993), we favored a dimeric HPX receptor. Additionally, because we show here that HL-60 cells do not express immunologically detectable LRP1, and given the known function of TfR1 in endocytosis, our study and other studies establish that there is a novel-LRP1-independent system for heme–HPX endocytosis and apo-HPX recycling. When HeLa cells, which also lack LRP1 (Mucci et al., 1995), are incubated with heme–HPX there is a rapid and extensive downregulation of surface TfRs (Taketani et al., 1990). Thus, evidence from these LRP1^−/−^ cells shows that LRP1 is not the sole HPX receptor (Hvidberg et al., 2005).

### 3.2 Cell biology of heme–HPX

#### 3.2.1 Evidence for heme–HPX co-localization with Tf and TfR1 supports HPX internalization and trafficking via clathrin-mediated Tf endocytosis in HL-60 cells

The route by which receptor-bound transferrin moves through cells is very well characterized, with rapid uptake into Rab5-positive endosomes (Grant and Donaldson, 2009; Mayle et al., 2012). These are an established population of early endosomes with cargo that return to the cell surface (Conner and Schmid, 2003; Mayle et al., 2012). Tf moves from the plasma membrane clathrin-coated pits to early endosomes, and after acidification and iron dissociation, apoTf is directed to recycling endosomes for trafficking to the cell surface and release. The endosomal location of ligands with Tf provides evidence that co-localized protein ligands are also being trafficked for recycling (Mayle et al., 2012), rather than via the degradative lysosomal route. Therefore, we investigated the trafficking of AF-labeled HPX with these protein markers, including Rab5 for early endosomes.

Binding of heme–HPX to the plasma membrane is extensive when HL-60 cells are incubated with heme–AF546HPX for 1 h at 4 °C (Figure 2E, surface binding). Upon cell warming, HL-60 cells take up both AF-Tf and heme–AFHPX complexes (Figure 2A) into early endosomes, identified by Rab5 staining (Figure 2C). In HL-60 cells, mHAF546–RbtHPX co-localizes with AF488Huholo-Tf (Figures 2A,B) and AF405TfR1 (Figures 2C,D), supporting that HPX is trafficked via early AF488Rab5-positive endosomes (Figures 2C,D) that return their cargo to the surface (Mayle et al., 2012). Surface TfR1 is downregulated in response to holo-Tf, which increases cell iron content, including in non-hematopoietic cells. Overnight incubation of HL-60 cells with holo-Tf significantly downregulated the surface expression of TfR1 in HL-60 cells (Figures 2E,F) and the surface HPX fluorescence, implicating TfR1s for surface binding of heme–HPX. When the cells were warmed to 37 °C for 10 min so that endocytosis takes place, heme–AF546HPX is detected intracellularly in vesicles, as indicated by punctate staining (Figures 2E,F). Thus, both HPX surface binding and endocytosis are significantly decreased by holo-Tf pre-incubation, strongly supporting that TfRs are involved in HPX endocytosis; and overall, implicating this pathway for the known apo-HPX recycling *in vitro* and *in vivo*.

There is some heterogeneity in the uptake of heme–AF546HPX and AF488 holo-Tf, including in cells where these ligands co-localize. HL-60 cells are small, and it is well documented that they can be heterogeneous in size (9–25 µm in diameter), appearance, and responses (Collins and Foster, 1983; Birnie, 1988; Salvioli et al., 2000). Their nucleus is large compared to the small volume of cytoplasm (Wang et al., 2015).

In conclusion, heme–HPX, which regulates cell iron homeostasis and proliferation/cell growth, is taken up by clathrin-mediated endocytosis and raises intracellular ferrous iron safely for the IRE/IRP system of translational regulation. Significantly, we have shown that heme–HPX binding and endocytosis initiate a series of events to regulate intracellular iron levels, not only limiting iron toxicity but also maintaining redox metal (*i.e.*, iron, copper, and heme) homeostasis in cells (Vanacore et al., 2019). In addition, HL-60 cells are a second LRP1^−/−^ cell line in which confocal microscopy has shown that heme–AF–HPX is taken up by endocytosis co-localized with an endosome marker (Smith, 2011). The established recycling of HPX is inconsistent with LRP1-mediated endocytosis that leads to the lysosomal degradation of HPX (Hvidberg et al., 2005). Support for an LRP1-independent uptake system for heme–HPX also comes from the evidence for endocytosis of heme–HPX in LRP1^−/−^ PEA 13 cells (Smith, 2011) and, as reported here, in HL-60 cells that lack LRP1. Overall, the ICC data presented here lay a foundation for more detailed investigations on HPX endocytosis.

#### 3.2.2 Hemopexin “interactome” in HL-60 cells

The well controlled heme-HPX affinity chromatography of cell extracts containing biotinylated surface proteins from undifferentiated human HL-60 cells, together with an unbiased analysis using LC-MS/MS, revealed some additional members of the HL-60 HPX “interactome,” providing novel and unique information on the HPX system in immune cells (Supplementary Table S1). As anticipated, TfR1 was the top target consistently and was shown to be derived from the cell surface of LRP1^−/−^HL-60 cells.

It is intriguing that three other proteins: pyruvate kinase and exocyst complex components 1 and 3, together with TfR1, are all linked with iron, although not yet mechanistically. Pyruvate kinase regulates glycolysis, and pyruvate kinase deficiency has been linked to iron overload. The energy of the cell is related to mitochondrial metabolism and is linked with other pathways, including glycolysis. In pyruvate kinase deficiency, there is ineffective erythropoiesis together with chronic hemolysis, leading to iron-loading anemia (Mojzikova et al., 2014). The exocyst complex has eight protein subunits and tethers secretory granules to the plasma membrane; it is also required for platelet granule secretion and receptor trafficking in platelets. Loss of this function may lead to thrombotic conditions detrimental to hemostasis (Walsh et al., 2021). Exocyst complex components 1 and 3 are essential for targeting exocytic vesicles to specific docking sites on the plasma membrane through interactions with GTPases. Such vesicles include Tf/TfR-containing recycling vesicles that are tethered and/or fused with the plasma membrane in concert with the exocyst tethering complex (Takahashi et al., 2012). Furthermore, extracellular vesicles termed exosomes, which are secreted by cells and contain cell constituents including DNA, RNA, and proteins, are eventually taken up by other tissue cells; cell function and behavior are affected and often signal pathology. It is conceivable that these proteins may represent a cell surface complex with the TfR1 homodimer that was not dissociated during SDS-PAGE. They are not detected with the TfR1 monomer.

In addition, PDCD6IP is part of the endosomal sorting complexes required for transport, *i.e.*, the ESCRT system, which plays a critical role in cells by pinching off small vesicles from larger membranes, thus contributing to membrane remodeling for endosomal sorting. It participates in both membrane scission of reverse topology budding and in multivesicular body formation. CPT1A is a rate-limiting enzyme of fatty acid oxidation that catalyzes the transfer of the long-chain acyl group in acyl-CoA ester to carnitine, allowing fatty acids to enter the mitochondrial matrix for oxidation. In addition, heme–HPX binds to a cell surface glycoprotein, CD 200 receptor 1, which is an OX-2 membrane glycoprotein from myeloid lineage cells that controls myeloid function in the spleen and placenta. This receptor is expressed in the liver, but at low levels. It inhibits the activation of human myeloid cells and may play a crucial role in skin wound healing by regulating the immune response to reduce scarring and decreasing mast cell degranulation and cytokine secretion response. Overall, the hemopexin interactome proteins are potentially novel components of HPX biochemistry and physiology, providing insight into the range of physiological functions for the HPX system.

#### 3.2.3 Immunocytochemical studies reveal co-localization of HPX with both TfR1 and LRP1 and also the separation of TfR and LRP1 trafficking in human hepatoma HepG2 cells

##### 3.2.3.1 Rationale for using human hepatoma HepG2 cells

Liver targeting of heme-HPX was observed after intravenous injection of heme–HPX in anesthetized rats (Smith and Morgan, 1978). Liver cells, presumably hepatocytes, take up heme-HPX and heme is released intracellularly while apo-hemopexin is recycled to the plasma. Intracellularly, heme is catabolized by heme oxygenase releasing the heme-iron that enters known iron pools with regulatory consequences and potentially as iron levels rise iron export. Consequently, the HPX system contributes to maintaining whole body iron homeostasis. Because of the clinical importance of HPX, it is essential to identify HPXRs expressed by liver cells. Nevertheless, research on non-hepatic cells, including HL-60 cells, has shown and continues to provide key information on other important aspects of the human HPX system, including HPX’s role in the immune system. Understanding hepatic heme regulation of plasma HPX levels (Foidart et al., 1982) and the hepatic clearance of heme is critical to fully understanding and improving targeted therapies to reduce heme toxicity. Thus, the dual approach here helps better define the entire process of heme clearance, including how multiple organs and cells coordinate protection from heme toxicity in both immune and hepatic cell models.

We first investigated the cell biology of HPX in human hepatoma HepG2 cells because they are an established model for human liver; however, they differ from hepatocytes because they are deficient in drug metabolism, lacking cytochrome P-450 and other key enzymes. In addition, being transformed cells, they have a fast growth rate compared with mature hepatocytes that normally do not proliferate. Furthermore, HepG2 cells have already been shown to endocytose transferrin and hemopexin, and these proteins co-localize in vesicles of the endocytotic pathway (Smith and Hunt, 1990).

The plasma membrane binding of heme–HPX in HepG2 cells is shown by co-localization with Na^+^ K^+^ ATPase (Supplementary Figures S2A and S2B). Upon cell warming, the heme complexes of both rabbit and human HPX are taken up into Rab5-positive early endosomes (Supplementary Figures S2C and S2D). Furthermore, Tf co-localizes with TfR1 in these cells (Figures 3A,B), showing that HPX is taken up into a pathway where it co-localizes with TfR1, following the classic clathrin-mediated pathway of endocytosis (Figures 3C–H). As anticipated, the type of heme molecule (*i.e.*, proto- or mesoheme) bound to HPX affects neither HPX endocytosis nor heme-dependent HMOX1 induction (see Figure 7).

To summarize, heme–HPX, like iron-Tf/TfR1, which regulates cell iron homeostasis and cell proliferation, is taken up by clathrin-mediated endocytosis; and raises intracellular ferrous iron safely for the IRE/IRP system of translational regulation.

**FIGURE 4:**
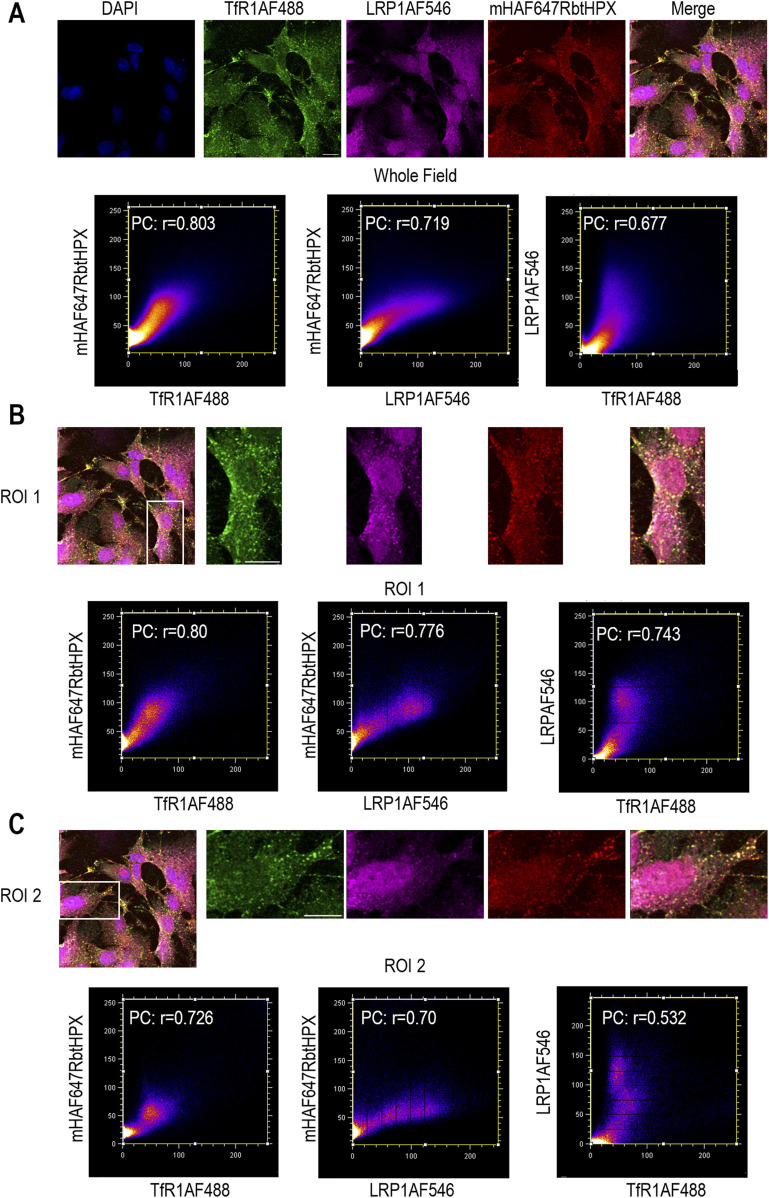
In HepG2 cells, ICC data support the intracellular co-localization of TfR1 and the low-density lipoprotein receptor 1 (LRP1) together with HPX. **(A–C)** Co-localization TfR1AF488 (green color), LRP1AF546 (magenta color), and mHAF647RbtHPX (red color) after 10 min incubation of HepG2 cells at 37 °C. These data, both whole field and two separate ROIs, support that HPX resides in one population of endocytotic vesicles together with both TfR1 and LRP1; however, there is also a significant population of vesicles predominantly with LRP1 but without TfR1. Cytofluorograms of the whole field and regions of interest indicate a positive PC; ROI 2, data. HepG2 scale bar = 15 µm.

We also investigated the cell biology of LRP1, which has not been defined; however, the generally accepted model is that after ligand binding to LRP1, the ligand–LRP1 complex is taken up into early endosomes (Laatsch et al., 2012), but while the ligands are targeted to the lysosomes, LRP1 recycles back to the cell surface. In LRP^+/+^HepG2 cells, ICC data show that HPX moves into two populations of endosomes (Figure 4). In one population, HPX resides with both LRP1 and TfR1. The relative distribution of HPX reveals a population of vesicles, where the signal from LRP1 predominates and is significantly enriched compared with TfR1. In a second population, HPX is present only with TfR1 (Figure 4, shown by the pixel distributions in the cytofluorogram: LRP1 v TfR1). Furthermore, some LRP1 is present in vesicles that lack both TfR1 and HPX. Thus, several distinct trafficking pathways for these two receptors exist in response to the ligand heme–HPX. Intriguingly, in LRP1^+/+^ HepG2 cells, AF–HPX trafficked in two populations of intracellular vesicles, either together with both TfR1 and LRP1 or with only TfR1. LRP1 was also detected, localized in a separate population of intracellular vesicles lacking both HPX and TfR1. These locations might represent, respectively, LRP1 in the TfR1-associated recycling pathway *en route* to the plasma membrane and alone in other intracellular pathways yet to be identified. The identification of TfR1 in the “hemopexin interactome” affinity isolations from extracts of HL-60 cells, together with our ICC data from HL-60 cells and also from HepG2 cells, both support and extend our previous studies, published in 1990, showing that HPX travels through HepG2 cells together with the Tf/TfR1 pathway via its clathrin-dependent pathway for endocytosis (Smith and Hunt, 1990).

#### 3.2.4 Liver targeting of HPX: evidence from immunocytochemical studies for HPX co-localization with TfR2 in human hepatoma LRP1^+/+^ HepG2 cells

Enigmatically, there are two transferrin receptors: TfR1 and its homolog, TfR2. Both bind diferric transferrin, but TfR1 binds this ligand far more tightly than TfR2 (Kleven et al., 2018). Thus, the interaction with iron–Tf differs between these two receptors. Nevertheless, the ectodomain of both TfRs are very similar (47% identity and 67% similarity in amino acid sequence (Kleven et al., 2018)), making it likely that TfR2 also binds heme–HPX. The differences between TfR1 and TfR2, namely, in their affinity for holo-Tf and in the cell types on which they are expressed, are important and have been linked to their different roles in iron homeostasis. TfR1 is expressed ubiquitously on cell surfaces, albeit at generally low levels, whereas TfR2 shows preferentially higher surface expression on reticulocytes, hepatocytes, placental cells, endothelial cells of the blood brain barrier. On the other hand, while TfR1 is expressed by all cells except mature erythrocytes and terminally differentiated cells, its homolog TfR2 is expressed predominantly in liver hepatocytes, but also in testes, erythroid cells, spleen, lung, and hepatoma lines, including HepG2 cells (Johnson and Enns, 2004).

**FIGURE 5:**
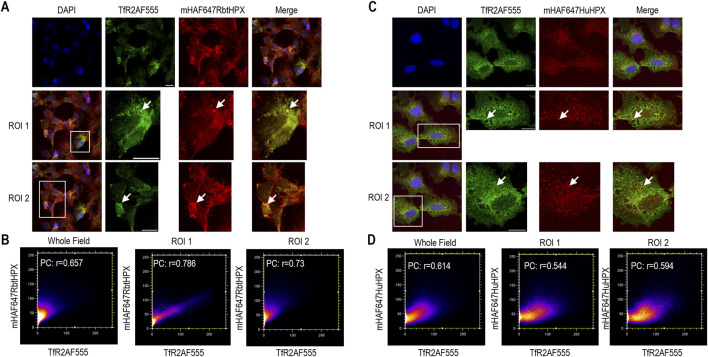
In HepG2 cells, heme complexes of both rabbit and human HPX are taken up and co-localize with TfR2, consistent with liver targeting of heme–HPX *in vivo*. **(A)** Co-localization of mesoheme–rabbit HPX (mHAF647RbtHPX, red color) with TfR2AF555 (green color) after 10 min of incubation of heme–HPX complexes with the HepG2 cells at 37 °C. **(C)** Co-localization of TfR2AF555 (green color) and mesoheme–human HPX (mHAF647HuHPX, red color) under the same conditions of incubation of heme–HPX complexes with the HepG2 cells. **(B, D)** Cytofluorograms of the whole field and regions of interest reveal a positive PC. The images were acquired using a high-performance 40×1.3 NA oil objective with a theoretical diffraction limit of ∼272 nm. Well-resolved features with strong co-localization, corresponding to the brightest and largest structures, are apparent in smaller punctate vesicles in several cells (see white arrows for examples). HepG2 scale bar = 15 µm.

TfR2 is expressed abundantly on hepatocytes (Worthen and Enns, 2014) and has been implicated in the maintenance of systemic iron homeostasis (Worthen and Enns, 2014). Thus, TfR2 would provide an entry point to the liver for the rapid and specific clearance of toxic heme through the endocytosis of heme-HPX under hemolytic conditions. We investigated whether heme-AF-HPX co-localizes with TfR2 in TfR2^+/+^HepG2 cells. ICC data show that both human and rabbit HPX rapidly co-localize and traffic with TfR2 in vesicles in HepG2 cells (Figures 5A–D). These data support that TfR2 is responsible for the known efficient targeting to the liver of intravenous heme-HPX (Smith and Morgan, 1979). Overall, the results of the ICC studies in human promyelocytic HL-60 cells and HepG2 cells provide relevant information supporting this intracellular route for HPX, after surface binding and uptake of heme-HPX complexes to TfR1 and potentially TfR2, followed by recycling of HPX.

### 3.3 Isolation and identification of heme–HPX binding proteins from two models of human liver cells: pilot studies

#### 3.3.1 Evidence for heme–HPX receptor-mediated heme transport in HepG2 cells and primary human hepatocytes using HMOX1 induction by heme–HPX

Several published studies on the HPX system/network with primary cells, *e.g.*, rat or human hepatocytes, have been published. Pioneering studies with the isolated perfused rat liver showed that rat, rabbit, and human HPX all deliver [^59^Fe]-labeled heme at similar rates to rat liver cells (Liem, 1976). Moreover, rat, rabbit, and human HPX all deliver heme effectively to freshly isolated rat hepatocytes (Smith and Morgan, 1981). This heme is utilized for the transcriptional regulation of HMOX1 (Sun et al., 2002), and heme catabolism releases iron, which acts in the translational regulation of key proteins involved in iron homeostasis [Casey et al. (1988) and see references in Rouault et al. (1990)]. This iron regulates several proteins involved in iron homeostasis at the translational level via the Fe/IRP/IRE system (Muckenthaler et al., 2008), including TfR1, which is downregulated when intracellular iron levels increase (Haile et al., 1989; Harford and Klausner, 1990). Because HL-60 cells respond to heme–HPX by inducing HMOX1 mRNA (Alam and Smith, 1989), consistent with heme-mediated regulation via release of Bach1 repression and activation of Nrf2 for HMOX1 transcription (Alam et al., 1999) and downregulation of TfR1 mRNA (Alam and Smith, 1989). Inhibition by cell-permeable iron chelators revealed that additional regulation required iron from rapid heme catabolism released intracellularly after incubation of heme–HPX in mouse Hepa cells (Sung et al., 2000). Using recombinant HPX, Hada et al. (2014) showed that heme–HPX endocytosis delivered heme to hepatoma cells (Hepa1c1c7), which downregulated Bach1, allowing Nrf2-mediated induction of HMOX1 transcription.

**FIGURE 6:**
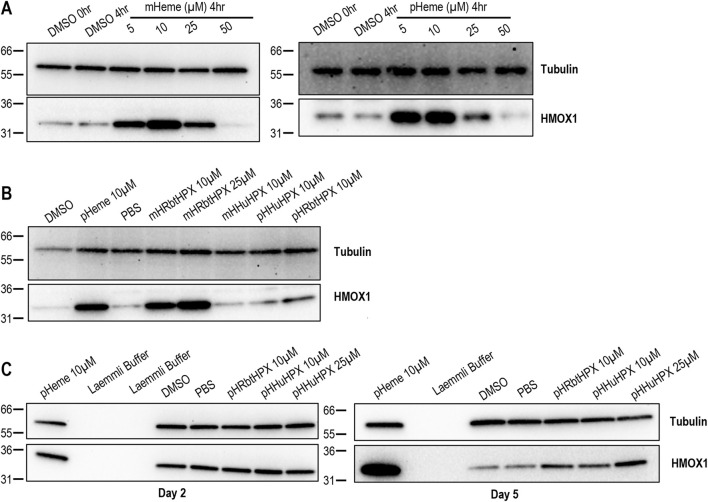
In HepG2 cells and primary human hepatocytes**,** HMOX1 induction requires heme, and its induction by heme–complexes of both rabbit and human HPX supports the uptake of heme–HPX for heme delivery to these cells. **(A, B)** Western blotting confirms that mesoheme (mH), proto-heme (pH), and heme–HPX complexes induce HMOX1 in HepG2 cells after 4 h of incubation at 37 °C. **(C)** On day 5 of culture, freshly plated human hepatocytes respond to heme–HPX complexes by inducing the HMOX1 protein. Data shown are representative of primary hepatocytes from liver donor A (see Supplementary Figure S2 for donor information); however, HMOX1 induction was apparent in hepatocytes isolated from three individual liver donors (data not shown).

Therefore, to establish the presence of functional receptors for heme–HPX uptake, we determined that heme–HPX induced HMOX1 in the HepG2 cells and also in the primary human hepatocytes. Both mesoheme and proto-heme, known HMOX1 inducers, significantly increased HMOX1 protein levels within 4 h (Figures 6A,B), as did both human and rabbit heme–HPX complexes. The slightly greater HMOX1 induction by “heme” compared with heme-HPX is because the rate of heme “diffusion” into cells is significantly faster than the gradual, relatively slow and safe heme delivery *via* heme-HPX endocytosis (Smith and Morgan, 1981). Nevertheless, the HMOX1 induction in response to HPX prevents heme toxicity *in vivo*.

Importantly, HMOX1 is also induced when primary human hepatocytes are incubated with proto-heme or proto-heme complexes of human HPX or rabbit HPX (Figure 6C). Furthermore, as anticipated, the response of primary human hepatocytes, *i.e.*, HMOX1 induction, was more extensive on day 5 compared with day 2 of culture (Figure 6C).

#### 3.3.2 Protein “HPX interactome” from HepG2 cells

**FIGURE 7:**
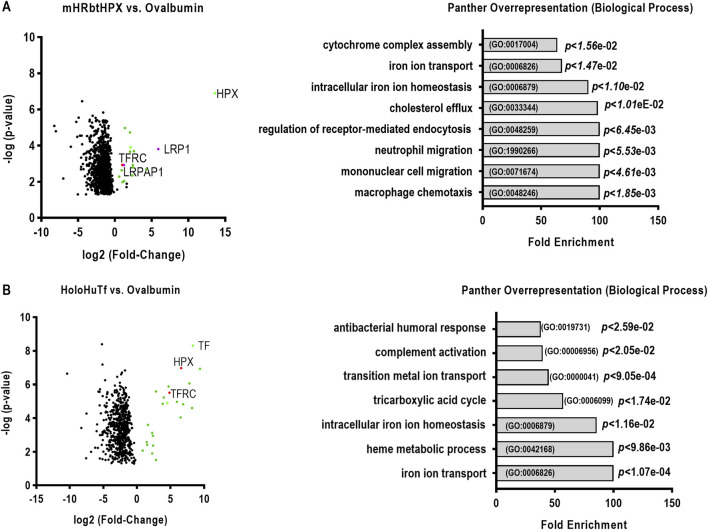
Analyses of ligand affinity isolates provide evidence that mesoheme–rabbit hemopexin (mHRbt HPX) binds both transferrin receptor 1 (gene name. TFRC) and LRP1. Volcano plots: Log_2_ fold change (mHRbtHPX v. negative control ovalbumin–Affi-Gel 15, **(A)**), and holoHuTf/ovalbumin-Affi-Gel 15 **(B)** represent the ratio of protein recovery from one biological experiment. Protein abundances were determined as defined using label-free quantification (LFQ) values measured from triplicate injections of each sample into the LC–MS/MS, considering these technical replicates as MaxQuant fractions (refer to Supplementary Methods section). MaxQuant LFQ protein abundances were used to calculate log_2_ protein ratios (mHRbtHPX/ovalbumin resin and HoloHuTf/ovalbumin resin). **(A)** mHRbtHPX (HPX, green dot), LRP1, and LRPAP1 (AKA “RAP,” magenta dot); TfR1 (red dot), and other significant proteins are shown (green dots). **(B)** HoloHuTf (TF green dot), TfR1 (TFRC red dot), and other significant proteins are shown (green dots). For further information on these proteins, see Supplementary Tables S1 and S2. The PANTHER overrepresentation data (RHS) reveal the most significant physiological processes after analysis for their overrepresentation in key biological processes using PANTHER GO-SLIM. The fold enrichment, Gene Ontology (GO) identifier (*y*-axis), and *p*-values are shown. A 100-fold enrichment means that a protein is present in the biological process 100 times more frequently than expected by random chance, indicating the strong degree of over-representation.

Affinity chromatography using heme–HPX Affi-gel 15 and whole-cell extracts of LRP1^+/+^ HepG2 cells isolated heme–HPX-binding proteins, which were identified using MS by peptide sequencing and the extent of coverage of the complete amino acid sequence. Data collected from one set of HepG2 isolates, analyzed in triplicate, allowed statistical analyses of the isolated proteins from both positive and negative control resins (ovalbumin Affi-Gel 15). Using PANTHER GO-Slim criteria (Figures 7A,B), the isolated proteins were assigned to biological processes. Both the gene and protein names of the holo-human Tf interactome (Table 2; Supplementary Table S2) and for mesoheme–rabbit HPX (Table 3; Supplementary Table S3) are provided. There is also a brief description of the protein function and, in the footnotes, a summary of the key biological processes in which they act.

**TABLE 2:** The holo-human transferrin “interactome” proteins isolated from HepG2 whole-cell extracts after binding to holo-human transferrin Affi-Gel 15, as a positive control for TfR1 (TFRC), compared with those from the negative control ovalbumin-Aff-Gel 15. The proteins are presented in order of significance. We used several online sources including the National Library of Medicine at NIH and GeneCards as resources for the summary of protein function. The biological processes associated with these proteins identified by PANTHER GO-Slim analysis include, but are not limited to, iron ion transport, heme metabolic process, intracellular iron ion homeostasis, tricarboxylic acid cycle, transition metal ion transport, complement activation, and anti-bacterial humoral immune response. The presence of Tf) in the eluates is likely due to leaching from the holo-human transferrin Affi-resin, which often occurs in this type of analysis and was not used in the PANTHER analyses.

Gene symbol	Protein name	Biological function
SMARCC1	SWI/SNF-related BAF chromatin remodeling complex subunit C1	Component of chromatin remodeling complex altering DNA-histone contacts
TF	Transferrin	Iron binding protein; transports iron in plasma
APOH	Apolipoprotein H	Component of plasma lipoproteins
CORO2A	Coronin 2A	Actin binding protein 2A (WD repeat)
IGHA1 and IGHA2	Immunoglobulin heavy constant ɣ1 and 2	Acts in antibody- and complement-dependent cytotoxicity
HPX	Hemopexin (human)	Binds heme, transports heme to the liver, and protects cells from oxidative stress
IGLC2 and IGLC3	Immunoglobulin lambda constant 2 and 3	Part of the constant region of Igʎ light chain antibodies produced by B lymphocytes
ACACA	Acetyl-CoA carboxylase 1	An enzyme catalyzing the first and regulatory step of fatty acid synthesis
TFRC*	Transferrin receptor 1	Cell surface receptor for iron uptake by binding holo transferrin
C2	Complement component 2	Key part of immune response to destroy bacteria and viruses
HPX	Hemopexin (rabbit)	Binds heme, transports heme to the liver, and protects cells from oxidative stress
COPS6	COP9 signalosome complex subunit	One subunit of the COP9 signalosome that regulates multiple signaling pathways
LTF	Lactoferrin	Member of transferrin iron-binding family present in secretory fluids and secondary granules of neutrophils
APOB	Apolipoprotein B	Component of LDL and transports cholesterol in circulation
PSMC6	Proteasome 26S subunit	Part of 26S proteasome complex to degrade ubiquitinated proteins
GGH	Gamma glutamyl hydrolase	Acts in the metabolism of folates and anti-folates
PPIF	Peptidyl-prolyl cis-trans isomerase F	Mitochondrial protein may assist in protein folding. Part of the mitochondrial permeability transition pore
RNF20 and RNG40	Ring finger protein 20 and 40	E3 ubiquitin-protein ligase involved in ubiquitination of histones and regulation of chromosome structure; putative tumor suppressor
BZW2	Basic leucine zipper and W2 domain containing protein 2	Ubiquitous, cytoplasmic, regulates eukaryotic translation initiation
TRIM21	Tripartite motif-containing protein 21	Involved in intracellular response to human pathogens e.g., invading viruses targeting them for degradation
KIF20B	Kinesin family member	Role in cytokinesis; ATP hydrolysis, located in several parts of cells
TRUB2	TruB pseudouridine synthase family member 2	Acts to modify uridine on some mitochondrial and tRNAs
IDH3A	Isocitrate dehydrogenase (NAD^+^) subunit alpha	Catalytic subunit of isocitrate dehydrogenase within mitochondria

**TABLE 3:** The heme–HPX interactome proteins from HepG2 whole-cell extracts after binding to mesoheme–rabbit HPX Affi-Gel 15 identified as significant by mass spectrometry. The negative control resin was ovalbumin-Affi-Gel 15. For the summary of protein function, we used several sources including the National Library of Medicine at NIH and GeneCards as resources. The proteins are presented from highest to lowest enrichment in terms of significance value. The biological processes identified with the PANTHER GO-Slim program for these protein targets include (highest to lowest enrichment), but are not limited to, macrophage chemotaxis, mononuclear cell migration, neutrophil migration, regulation of receptor-mediated endocytosis, cholesterol efflux, intracellular iron ion homeostasis, iron ion transport, and cytochrome complex assembly. The presence of HPX is considered due to leaching of the protein from the beads, which often occurs in this type of analyses, and proteins, therefore, were not included in the PANTHER analyses.

Gene symbol	Protein name	Biological function
HPX	Hemopexin	Binds heme, transports heme to the liver, and protects cells from oxidative stress
LRP1*	Low-density lipoprotein receptor 1	Scavenger receptor with a multitude of ligands, some of which are non-native and target them to lysosomes
PSMB8	Proteasome subunit βtype-8	Provides instructions for making immunoproteasome, involved in antigen processing and the inflammatory response
TRUB2	TrubB pseudouridine synthase	Enzyme that catalyzes the formation of pseudouridine in tRNA molecules; associated with sideroblastic anemias and myopathy
PDF	Peptide deformylase	Enzyme associated with Sennetsu fever and bacterial infectious diseases
CTSH	Cathepsin H	Lysosomal cysteine protease, degrades intracellular proteins
ENOPH1	Enolase-phosphatase I	Bifunctional enzyme, tumor promoting properties in breast cancer, activates the NF-κB pathway
TRIM21	Tripartite motif-containing protein 21	Cytosolic antibody receptor and E3 ubiquitin ligase, destroys pathogens *via* antibody-dependent mechanisms
CLINS1A	Chloride nucleotide-sensitive channel 1A	Functions in multiple regulatory pathways, together with cytosolic proteins, including mRNA processing and mRNA splicing
NUFIP2	Nuclear FMR1 interacting protein 2	Localized to nucleoplasm, cytoplasmic stress granules; RNA binding protein; prognostic marker in pancreatic adenocarcinoma
RPS4Y1	Ribosomal protein S4	Subunit of 40S subunit; RNA binding rRNA binding; binds to numerous cytoplasmic proteins and regulates small nuclear ribonucleoprotein biosynthesis, platelet activation, and cytoskeletal organization
DEPDC1	DEP domain containing 1	Associated with tumors; essential for bladder cancer cell proliferation, promotes JNK-dependent degradation of BCL-2 family protein MCL1
APOAP1	Apolipoprotein A-1	Primary protein of high-density lipoprotein HDL in blood
LRPAP1	LRP1 receptor-associated protein (RAP)	Chaperone regulating activity of LRP1 by inhibiting ligand binding, prevents LRP1 from reaching cell membrane; role in microglial function and amyloid β aggregation in neurological diseases
SRP14	Signal recognition particle 14	Component of the SRP complex for co-translational targeting of secretory and membrane proteins in the ER
TFRC*	Transferrin receptor 1	Required for iron uptake into cells by endocytosis
FBX02	F-Box protein 2	Part of ubiquitin protein ligase complex
COA6	Cytochrome c oxidase subunit family member	May act as mitochondrial complex VI assembly factor, possibly a redox protein
LGALS3	Galectin 3	Carbohydrate binding protein

Both LRP1 and TfR1 were isolated and unequivocally identified in the HepG2 extracts from the mesoheme–rabbit Affi-Gel 15 (Figure 7A; Table 3; Supplementary Table S3). In addition, the following proteins are present: a proteasome subunit of β-type B, PSMB8, which helps form the immunoproteasome involved in antigen processing for the inflammatory response; TRUB2, a synthase that adds pseudouridine to tRNA molecules; PDF, an enzyme associated with bacterial infectious diseases; ENOPH1, which activates the NF-κB pathway; and TRIM21, which helps destroy pathogens. Furthermore, the LRP-related protein-associated protein 1, LRPAP1, known as receptor-associated protein, RAP, was identified, which is a chaperone that helps transport LDL receptors to the cell membrane and facilitates their proper folding and localization. RAP may limit amyloid-β clearance and has been linked to dementia. The biological processes in which all of these and additional proteins are linked include macrophage chemotaxis, mononuclear cell and neutrophil migration, regulation of receptor-mediated endocytosis, cholesterol efflux, intracellular iron homeostasis and iron-ion transport, and cytochrome complex assembly (Figure 7A).

As a positive control for TfR1 (gene name TFRC), we investigated the HepG2 proteins that either bound specifically to holoHuTf beads or were enriched compared with the eluates from the negative control resin, ovalbumin Affi-Gel 15 (Figure 7B; Table 2; Supplementary Table S2). TfR1 and additional proteins of interest in this holo-human Tf “interactome” were linked with immune system defense, such as complement. Unexpectedly, HPX was also identified, suggesting that HPX, when binding heme and holo-transferrin may have an affinity for each other. Apolipoprotein H (APOH) was found to be a protein component of plasma lipoproteins involved in lipid metabolism, coagulation, and hemostasis. SMARCC1, a member of the SW1/SNF family, was also isolated, which acts as a helicase and ATPase. SMARCC1 may regulate gene transcription by altering chromatin structure. Coronin 2A (CORO2A) is a WD repeat protein family member that facilitates the formation of multi-protein complexes involved in a variety of cellular processes, including actin microfilaments, endosomal fission, and clathrin-dependent cargo uptake. Immunoglobulin constant 2 (IGLC6), a secreted immunoglobulin, acts during the effector phase of humoral immunity to aid in the removal of bound antigens. The calcium cation antiporter (CaCA) is vital for calcium homeostasis and thus needed for defense and development. In addition, lactotransferrin (LTF) was identified, which binds iron thus limiting bacterial growth. The biological processes associated with these human liver proteins identified by PANTHER GO Slim analysis, include but are not limited to, iron ion transport, heme metabolic process, intracellular iron ion homeostasis, tricarboxylic acid cycle, transition metal ion transport, complement activation, and anti-bacterial humoral immune response. Interestingly, the function of the affinity-isolated proteins reveals that both HPX and Tf are linked with the protective immune responses and iron transport.

#### 3.3.3 Affinity isolation and identification of heme–HPX binding proteins of the hemopexin interactome from primary human hepatocytes

A comparison of the transcriptome from human liver-like models, including hepatocyte-like cells and primary human hepatocytes (Ardisasmita et al., 2022), revealed that, at that time, most liver cell models were incomplete compared with the human hepatocytes. Induced human pluripotent stem cell-derived liver models, developed meanwhile, remain challenging due to technical issues with differentiation and stable expression of cytochrome P-450 enzymes (Graffmann et al., 2022). In this study, we used primary human hepatocytes, which express the heme-proteins, cytochrome P-450s, to compare the HPX “interactome” proteins with those from one accepted model, the hepatoma HepG2 cells.

Images of primary hepatocytes during culture and the key information on liver donors (kindly provided by BioIVT, Kansas City, KS, United States) are presented (Supplementary Figure S3). The cells show features of a confluent monolayer of mature primary hepatocytes with the expected “cobblestone” morphology covering the plate surface. Because HPX binding proteins were being isolated from human livers and depend in part upon the medical history and health status of the liver donor, we did not accept livers from donors with a high basal metabolic index (BMI). Viral infections, including COVID-19, can increase the iron load of liver cells via TfR1 uptake that might downregulate TfR2 (Suriawinata and Mehta, 2023). However, we were not able to avoid livers from donors who had been infected with Epstein Barr virus (EBV), which is highly prevalent in the population of the United States, infecting more than 95% of American adults (Bjornevik et al., 2022).

For this pilot study, we used data from a single set of hepatocytes obtained from one human liver donor. Nevertheless, the data collected allow statistical analyses of the isolated proteins present in the Affi-Gel 15 isolates from our positive resins and three negative control resins (Table 4). These proteins are identified using MS, as previously described for isolates of the HL-60 and HepG2 cells. Using Scaffold software, the raw data were exported into an Excel sheet after setting the false discovery rate to 1.0% (FDR), the minimum number of peptides to 2, and the peptide threshold to 1.0% FDR. Proteins were then filtered by setting the proteins isolated from the control Affi-Gels to zero (underivatized, mouse IgG, and ovalbumin) to determine the specific proteins in our HHPX and HolohuTf Affi-Gel isolates. Holo human Tf affinity isolates were used as a positive control for TfRs; the proteins from human liver in the holo-human transferrin “interactome” (Table 4) included TfR1 but not LRP1. Input data (48 proteins) analyzed with the PANTHER GO Slim (Mi et al., 2019) are summarized in Table 4. Complement factor 1 proteins (CB\FB), among other components of the immune system, were abundant, more so than with the HepG2 cell proteins, as were prekallikrein B1, which is needed for thrombin production for coagulation, and SERPINA5, which inhibits protein kinase C in blood coagulation. Several proteins are part of immune system activation (complement activation, antimicrobial humoral response, immunoglobulin-mediated immune response, and leukocyte-mediated immunity) and receptor-mediated endocytosis.

**TABLE 4:** The holo-human transferrin interactome proteins isolated from detergent extracts of primary human hepatocytes using affinity isolation with holo-human transferrin Affi-Gel 15 identified as significant by mass spectrometry. The negative control resins were underivatized resin and ovalbumin- and mouse IgG-Affi Gel 15. For the summary of protein function, we used several sources including the National Library of Medicine at NIH and GeneCards as resources. The proteins are presented from highest to lowest enrichment in terms of significance value. Analysis using the PANTHER GO Slim program to reveal the biological processes with which these proteins are involved reveal immune system activation (complement activation, antimicrobial humoral response, activation of immune response, immunoglobulin mediated immune response, leukocyte-mediated immunity, and receptor-mediated endocytosis, among others. Data from primary hepatocytes of a second human liver contained 70% of these proteins (CFI, IGHG1, KLKB1, APOA2, APOH, C2, FETUB, SERPINA5, TFRC, CPB2, IGFBP3, and PLG). Additional proteins (gene names) include HRG, TF, APOA2, CFB, IGHG4, ITIH4, and IGHV3OR16-9. Most of these additional proteins also function in the immune and vascular systems, specifically in the complement pathways in lipid transport; and ITIH4 may act in liver regeneration or development possibly as a response to surgical trauma.

Gene symbol	Protein name	Biological function
CFI	Complement factor 1	A trypsin-like serine protease that is part of the complement system regulating the immune response
IGHG1	Immunoglobulin heavy constant ɣ1	Constant region of antibodies secreted by B-lymphocytes that participate in the recognition phase of humoral immunity to eliminate antigens
KLKB1	Prekallikrein B1	A serine protease and anti-coagulant secreted by the liver into the blood for thrombin production *via* the protein C system
APOH	Apolipoprotein H	B to negatively charged phospholipids in lipoproteins: acts in lipoprotein metabolisms, hemostasis, and antiphospholipid antibody production
SERPINA5	Protein C inhibitor	Serpin family member, a serine protease inhibitor inhibits activated protein C in blood coagulation
FETUB	Fetuin-B	A cysteine protease inhibitor that acts in the regulation of insulin and hepatocyte growth factor receptors and response to systemic inflammation
ITIH4	Inter-alpha-trypsin inhibitor heavy chain H4	Type II acute phase protein; produced by liver, a protease inhibitor functioning in inflammatory response to trauma
APOA2	Apolipoprotein A-II	Binds to lipids and stabilizes HDL
IGHG4	Immunoglobulin heavy constant gamma-4	Acts in antibacterial humoral response and activation of classical complement pathway
APOD	Apolipoprotein D	Lipocalin family member; with lecithin and cholesterol acyl transferase in a macromolecular complex for bilin transport and other ligands
C2	Complement C2	Part of the classical pathway of complement for immune response
TFRC*	Transferrin receptor 1	An integral plasma membrane receptor for iron and vital for iron uptake into cells from transferrin by endocytosis
IGHA1	Immunoglobulin heavy constant alpha 1	Part of the constant region of immunoglobulin A heavy chain that is crucial for immune function in mucous membranes
IGKC	Immunoglobulin kappa constant	Antibody produced by B lymphocytes to eliminate antigens
PLG	Plasminogen	Liver precursor of the enzyme plasmin that breaks down fibrin needed for blood clots
CFB	Complement factor B	Regulates cell senescence and interacts with alternative complement pathway for the immune system
IGFBP	Insulin-like growth factor	Forms a ternary complex and in plasma prolongs half-life of insulin-like growth factors regulating their interaction with growth factors
CFD	Complement factor D	Regulates collagen type-1 expression, migration of cell such as fibroblasts for tendon repair and healing
CBP2	Cbp2	Serine protease inhibitor, binds collagen, may act in collagen biosynthesis
ENPP2	Autotaxin	A secreted lysophospholipase D producing a signaling molecule, lysophosphatidic acid in extracellular fluids required by activated platelets

Unexpectedly, fewer specifically bound proteins were isolated from the proto-heme–human HPX Affi-Gel 15 (Table 5; than from the mesoheme–rabbit HPX Affi-Gel 15 (Table 6). Interestingly, angioprotein-like protein 3 (ANGPTL3) bound to the human heme–HPX resin. After cleavage, the N-terminus of this liver protein acts in lipid metabolism by controlling the levels of several lipid molecules and overall maintaining cholesterol balance, while the C-terminal chain is involved in angiogenesis (*i.e.*, the growth of new capillaries from blood vessels). Furthermore, LRP1 was eluted from both human and rabbit heme–HPX Affi-gels; however, TfR1 was isolated only from the rabbit HPX Affi-Gel. Both HPX Affi isolates contained complement factor 1, which controls complement pathways and thus immune responses, and Prekallikrein B1, needed for thrombin production and coagulation.

Overall, the biological processes in the human liver where proteins of the human HPX interactome are involved include regulation of lipid metabolism (regulation of lipase activity, cholesterol homeostasis), receptor-mediated endocytosis, immune system activation (complement activation, antibacterial humoral response, defense response to bacterium), and regulation of coagulation and wound healing. Additional identified processes include hemostasis, blood coagulation, regulation of body fluids (*i.e.*, kidney function), regulation of iron ion transport, and the antibacterial humoral response.

**TABLE 5:** The heme–human HPX interactome proteins isolated from detergent extracts of primary human hepatocytes after specifically binding to proto-heme–human HPX Affi-Gel 15 and identified as significant by mass spectrometry. The negative control resins were underivatized resinand ovalbumin- and mouse IgG-Affi Gel 15. We used several sources including the National Library of Medicine at NIH and GeneCards as resources forthe summary of protein function. The proteins are presented from highest to lowest enrichment in terms of significance value. Analysis using PANTHERGO Slim revealed the biological processes with which these proteins are involved: regulation of lipid metabolism (regulation of lipase activity andcholesterol homeostasis); receptor-mediated endocytosis; and immune system activation (complement activation, antibacterial humoral response, and defense response to bacterium).

Gene symbol	Protein name	Biological function
CFI	Complement factor I	Serine protease that is essential for immune system function because it controls all the complement pathways
IGHG1	Immunoglobulin heavy constant gamma 1	Constant region of antibodies secreted by B-lymphocytes that participate in the recognition phase of humoral immunity to eliminate antigens
KLKB1	Prekallikrein B1	A serine protease and anti-coagulant secreted by the liver into the blood for thrombin production *via* the protein C system
LRP1*	Low-density lipoprotein receptor 1	Scavenger receptor with several hundred ligands, some non-native proteins
ANGPTL3	Angiopoietin-like protein 3	After cleavage, the N-terminal peptide acts in lipid metabolism and the C-terminal peptide in angiogenesis producing new blood capillaries

**TABLE 6:** The heme–rabbit HPX interactome proteins from extracts of primary human hepatocytes after specifically binding to mesoheme–rabbit HPX Affi-Gel 15 identified as significant by mass spectrometry. The negative control resins were underivatized resin and ovalbumin- and mouse IgG-Affi Gel 15. From the PANTHER GO Slim analyses, the biological processes included are as follows: regulation of coagulation, regulation of wound healing, complement activation, hemostasis, blood coagulation, regulation of body fluid levels, iron ion transport, and the antibacterial humoral response.

Gene symbol	Protein name	Biological function
CFI	Complement Factor I	Serine protease that is essential for immune system function because it controls all the complement pathways
IGHG1	Immunoglobulin heavy constant gamma 1	Constant region of antibodies secreted by B-lymphocytes that participate in the recognition phase of humoral immunity to eliminate antigens
KLKB1	Prekallikrein B1	A serine protease and anti-coagulant secreted by the liver into the blood for thrombin production via the protein C system
APOH	Apolipoprotein H	In plasma binds to negatively charged phospholipids so in plasma lipoproteins acts in lipoprotein metabolisms, hemostasis and antiphospholipid antibody production
SERPINA5	Protein C inhibitor	Serpin family member, a serine protease inhibitor inhibits activated protein C in blood coagulation
FETUB	Fetuin-B	A cysteine protease inhibitor that acts in regulation of insulin and hepatocyte growth factor receptors and response to systemic inflammation
ITIH4	Inter-alpha-trypsin inhibitor heavy chain H4	Type II acute phase protein; produced by liver, a protease inhibitor functioning in inflammatory response to trauma
APOA2	Apolipoprotein A-II	Binds to lipids and stabilizes HDL
IGHG4	Immunoglobulin heavy constant gamma-4	Acts in antibacterial humoral response and activation of classical complement pathway
APOD	Apolipoprotein D	Lipocalin family member; found with lecithin and cholesterol acyl transferase in a macromolecular complex for bilin transport and for other ligands
C2	Complement C2	Part of the classical pathway of complement for immune response
TFRC*	Transferrin receptor 1	An integral plasma membrane receptor for iron and vital for iron uptake into cells from transferrin by endocytosis
IGHA1	Immunoglobulin heavy constant alpha 1	Part of the constant region of immunoglobulin A heavy chain that is crucial for immune function in mucous membranes
IGKC	Immunoglobulin kappa constant	Antibody produced by B lymphocytes to eliminate antigens
PLG	Plasminogen	Liver precursor of the enzyme plasmin that breaks down fibrin needed for blood clots
CFB	Complement factor B	Regulates cell senescence and interacts with alternative complement pathway for the immune system
LRP1*	Low density lipoprotein receptor 1	Scavenger receptor with several hundred ligands, some non-native proteins
HRG	Histidine-rich glycoprotein	Multi-ligand binding protein; adapter protein in pathogen clearance, cell chemotaxis coagulation, fibrinolysis among other functions
F12	Coagulation factor XII	A zymogen in plasma, activated to a serine protease that initiates the coagulation cascade
SERPINA1	Alpha-1 antitrypsin	A serine protease inhibitor protects lung cells against damage by leukocyte elastase.
SHBG	Sex hormone- binding globulin	Transports sex hormones into blood regulating access to target tissues
CP	Ceruloplasmin	Moves iron from organs into blood via loading transferrin
LUM	Lumican	Extracellular matrix small leucine rich protein regulates collagen and hence tissue repair
HP	Haptoglobin	Plasma protein that binds hemoglobin preventing toxicity
HPR	Haptoglobin related protein	Binds Hb in plasma as part of innate immune system in HDL particles against protective in sleeping sickness and ahaptoglobinemia
IL6ST	Interleukin 6 cytokine family signal transducer	Key component of cytokine receptor complex for signaling pathways shared by many cytokines including IL6 LIF and OSM
APOC1	Apolipoprotein C1	Primarily a liver protein, activated when monocytes differentiate into macrophages with central role in HDL and VLDL metabolism
CDH5	Cadherin 5	Ca-dependent cell adhesion protein; role in endothelial adherens junction assembly and maintenance.
PRCP	Prolyl carboxypeptidase	Precursor of lysosomal prolylcarboxy peptidase activates factors that regulate cell growth, angiogenesis and electrolyte balance
MEGF10	Multiple Epidermal Growth Factor-like Domains Protein 10	A membrane receptor (for C1q) in macrophages and astrocytes for phagocytosis of apoptotic cells and amyloid-beta uptake in brain
FCN3	Ficolin 3	Has lectin activity and can activate initial triggering of the complement pathway
H6PD	Hexose-6-phosphate Dehydrogenase	Catalyzes first steps of pentose phosphate pathway within ER generating NADPH, a vial reducing agent for cells
C1QB	Complement C1q subcomponent subunit B	With proenzymes C1r and C1s, acts as part of the complement C1 complex recognizing immune complexes
PLTP	Phospholipid transfer protein	Acts to transfer phospholipids between different lipoprotein particles in the blood and regulating cholesterol metabolism. A P53 target gene with roles in ferroptosis and in growth suppression in cancer
SERPING1	C1 inhibitor	A serine protease inhibitor made in liver that regulates the complement cascade via regulating bradykinin
SELENOP	Selenoprotein P	Plasma protein and key player in selenium metabolism, transporting Se to brain and testis as a key part of antioxidant defense by Se
SERPINA4	Kallistatin	Protein that signals through 4 receptors including LRP6 integrinβ3, nucleolin and Kruppel-like factor 4 and inhibits activities of tissue kallikrein
DBH	Dopamine β-hydroxylase	Oxidizes dopamine generating norepinephrine a hormone and main neurotransmitter in the sympathetic nervous system
CST6	Cystatin E/M	A cysteine protease inhibitor in the regulation of epidermal cornification
AOC3	Amine oxidase, copper containing 3	A type 1 membrane bound glycoprotein catalyze the oxidation of endogenous amines including histamine or dopamine with roles during inflammation and associated with many vascular diseases
APOB	Apolipoprotein B	Ligand for LDL receptor and major apolipoprotein of circulating chylomicrons and low density lipoproteins
C1QC	Complement C1q C chain	Key component of classical complement pathway in the immune system -activates the complement cascade
F11	Coagulation factor XI	A zymogen in blood activated for the blood coagulation cascade forming blood clots upon injury and activating other coagulation factors (factor IX)
SERPINA3	Alpha-1-antichymotrypsin	A protease inhibitor released into plasma during inflammation; protects tissues against damage by inhibiting serine proteases and chymases in neutrophils and mast cells
GPLD1	Glycosylphosphatidylinositol specific phospholipase D1	Degrades glycosylphosphatidylinositol anchors releasing proteins from the cell membrane into bloodstream
SFTPB	Surfactant protein B	Component protein of surfactant, a mixture of fats and proteins that lines healthy lung tissue facilitating breathing and oxygen exchange
F2	Prothrombin (AKA coagulation factor II)	The precursor for thrombin crucial for blood clotting at injury sites by converting fibrinogen to fibrin
AGT	Angiotensinogen	A liver precursor for needed angiotensin II with a crucial role in controlling blood pressure, body fluid and electrolyte balance.
CA1	Carbonic anhydrase 1	A zinc metalloenzyme that catalyzes the reversible hydration of carbon dioxide with water into bicarbonate and protons to form fluids like saliva and cerebrospinal fluid

The affinity of human HPX for TfRs may be lower than that of rabbit HPX. Nevertheless, the most abundant proteins in both the rabbit and human HPX Affi-isolates are the same and included complement factor 1, immunoglobulin heavy constant gamma 1, and prekallikrein B1, along with several additional high-abundance proteins such as inter-alpha-trypsin inhibitor heavy chain 4, which functions in the inflammatory response to trauma, together with several additional proteins involved in the immune response. In addition, proteins identified were involved in bilirubin transport, a key liver function; plasminogen, for blood clotting; and lumican, which links with collagen and tissue repair. Haptoglobin and haptoglobin-related protein (HPR) help protect against hemoglobin toxicity in hemolytic conditions, while IL6CT is part of a cytokine receptor complex for signaling pathways shared by several cytokines, including interleukin 6, a key component driving inflammation. Selenoproteins form a structurally diverse group of antioxidant enzymes. Selenoprotein P, which maintains a reservoir of selenium in plasma and also transports selenium to the brain and other tissues, was detected in the rabbit mesoheme–HPX Affi-eluate.

Receptor associated protein (LRPAP1) for newly synthesized LRP1, which also functions as an antagonist by interfering with the binding of this receptor to its multitude of ligands and may affect receptor folding (Marakasova et al., 2021). RAP was detected in the mesoheme–rabbit Affi-Gel isolates from HepG2 cells. Therefore, we investigated which human hepatocyte Affi-Gel-isolated protein levels were decreased by the presence of RAP (500 nM). RAP significantly decreased by 50% the interaction of proto-heme–human HPX with LRP1 and also of mesoheme–rabbit HPX with TfR1.

The MS analysis of the human hepatocyte HPX-Affi gel data are available through the data repository, the ProteomeXchange; please follow the link below. Furthermore, additional analyses and Excel summaries using the PANTHER Classification System (pantherdb.org) program and the GO-Slim Biological Process were used for statistical overrepresentation. The mass spectrometry proteomic data have been deposited in the ProteomeXchange Consortium *via* the PRIDE [1] partner repository with the dataset identifier PXD060789 and 10.6019/PXD060789.

Unlike TfR1, TfR2 is not downregulated in response to iron due to a lack of iron-regulator regions in mRNA (Testi et al., 2019). Thus, TfR2 would enable the rapid and specific hepatic clearance of toxic heme from plasma by HPX in a variety of clinical hemolytic conditions, where iron homeostasis at both cellular and systemic levels is in flux. Our model for TfR2 in heme–HPX endocytosis and heme clearance to the liver is based on preliminary data. Nevertheless, the data presented here overall extend the importance of HPX and heme clearance from biological fluids, including plasma, and implicate a previously unanticipated and potentially key role for the HPX system in the regulation of many aspects of heme-linked iron homeostasis in health and disease. Heme–HPX uptake via TfR2, which is enriched on precursor red blood cells, may also occur during some stages of hemolysis.

### 3.4 Evidence that hemopexin like transferrin is a heparin-binding protein: Implications for interactions of heme–HPX complexes with hepatic heparan sulfate proteoglycans and surface proteins

In mice, the protective role of HPX in inflammation has been linked to midkine, a cytokine and heparin-binding growth factor (Fagoonee et al., 2006). Heparin is an anti-coagulant and is also structurally related to the glycosaminoglycan (GAG) chains of sulfated proteoglycans that often associate with receptors at liver cell surfaces (Hu and Regoeczi, 1992). Heparin affinity chromatography revealed that while apo-Tf and holo-Tf bind to heparin leading at pH 7.4, only apo-Tf binds at acidic pH equivalent to the mature endosome. Thus, a model was proposed whereby holo-human-Tf bound to surface TfRs via a TfR-associated HSPG (Regoeczi et al., 1994).

**FIGURE 8:**
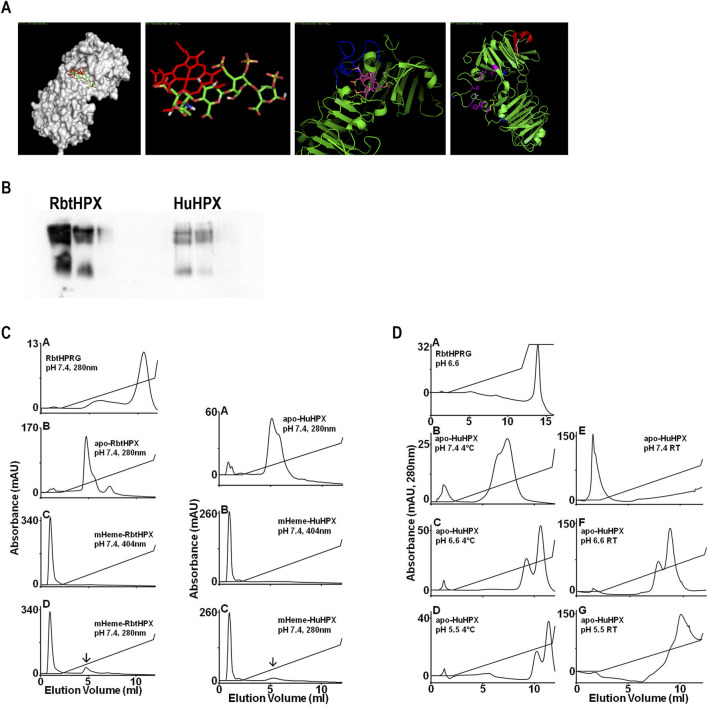
Heparin affinity chromatography to model the interaction of hemopexin with receptors and HSPGs. Docking models reveal that the proposed heparin binding site overlaps withthe heme binding site. Both human and rabbit apo-hemopexin bind to heparin at acidic pH ,representing the maturing endosome. **(A)** From left to right**:** Docked heparin on the surface of the crystal structure of the heme–hemopexin complex. The AutoDoc Vina program predicted one heparin binding site (green) on HPX overlapping with the heme (red) binding site, and the heme and heparin overlay reveals that these ligands of hemopexin occupy the same space. Docked heparin on the crystal structure of the heme–hemopexin complex. Linker peptide between the two N- and C_ beta-propeller domains of hemopexin. The heparin binding motif is depicted in blue. One heme coordinating histidine is located on this peptide. In addition, hydrogen bonds (yellow dashed lines) are shown. Depiction of the side chains of amino acid asparagine residues to which carbohydrate chains of hemopexin are bound. They cluster and reside on the opposite side of the molecule from the receptor binding site [Jen 14 epitope (Morgan et al., 1993)] in red. **(B)** Native gel electrophoresis of rabbit and human HPX reveals, based on their migration, three similarly charged species for both HPX congeners. **(C)** Affinity chromatography of apo- and heme-bound rabbit HPX (top to bottom) and similarly of apo- and heme-bound human HPX on HiTrap Heparin HP. Top: the elution profile of the heparin-binding protein, HPRG (200 µg), in sodium phosphate buffer, pH 7.4, with a salt gradient. The elution profiles of apo-rabbit (Rbt) HPX (200 µg) and mesoheme–Rbt HPX (90% saturated; an arrow shows the ∼10% apo-hemopexin), as expected for this saturation. The elution of rabbit (Rbt) HPRG occurs at 10.6 mL of the gradient, *i.e.,* 886 mM NaCl (conductivity 45.7 mS/cm); apo-rabbit HPX at pH 7.4 occurs at 4.75 mL of the NaCl gradient, *i.e.,* 270 mM NaCl (conductivity 2.02 mS/cm); and apo-human HPX (HuHPX) at pH 7.4 at RT elutes at 1.6 mL, corresponding to 0 mM Nacl (conductivity 1.8 mS/cm). The NaCl gradient begins at 2 mL elution volume. **(D)** Heparin affinity chromatography of apo-human HPX at neutral to acidic pH representing the maturation of endosomes. Affinity chromatography resolves two forms of HPX with different affinities for heparin; however, these were not resolved under either reducing denaturing or non-reducing conditions using electrophoresis (data not shown).

Therefore, we investigated the interactions of heparin with apo-HPX and heme–HPX as a model for HPX–surface HSPG interactions. First, we show that in the HPX molecule, a positive potential was indicated by highly conserved arginine and histidine residues in the heme pocket, which might provide a region for an electrostatic interaction with heparin. With one heparin repeat as the small molecule, only one heparin binding site was predicted using the AutoDock VINA program (Trott and Olson, 2010). Because the entire HPX molecule was used as the target, there was no bias. Intriguingly, heparin is depicted as bound to HPX in a region overlapping with part of the site where heme binds in the crystal structure (Paoli et al., 1999) of the heme–HPX complex (Figure 8A).

To assess directly whether there was a physical interaction between HPX and heparin we used affinity chromatography on Hi-Trap heparin-HP columns at pH 7.4, 6.6, and 5.5. The HPX elution profiles were compared with those of a known heparin-binding protein, rabbit HPRG. HPRG binds low molecular weight heparin with 0.3 µM affinity with a titration curve midpoint of pH 6.8 (Borza and Morgan, 1998). HPRG bound to the heparin-HP column and was eluted by 886 mM NaCl (rabbit HPRG); furthermore, both apo-rabbit HPX and human HPX also bind at pH 7.4, Figure 8C). Consistent with the prediction of overlapping heme and heparin binding sites on HPX at neutral pH, apo-HPX isolated from human and rabbit plasma binds to heparin, but heme–HPX complexes do not. Importantly, both human and rabbit apo-HPX bind to heparin effectively at acidic pH, which would occur in endosomes upon their maturation. Human apo-HPX required a slightly higher concentration of NaCl to elute from the resin than apo–rabbit HPX, supporting tighter binding, perhaps due to the additional O-linked carbohydrate chain on the N-terminal threonine. The number of histidine residues or basic amino acids, such as lysine, known to predominate in heparin binding sites is similar in these congeners (Ori et al., 2009). In addition, native gel electrophoresis revealed three differently charged species for both human and rabbit HPX, with similar migration patterns (Figure 8B). HPX levels in plasma range from 0.4 to 1.5 mg/mL and average approximately 15.3 μM (Smith and McCulloh, 2016); these are ∼14-fold higher molar concentrations, than heparin in plasma [only 1–5 μg/mL; 0.22–1.11 μM (Engelberg, 1961)].

Heme is bound tightly to HPX at pH 7.4 but dissociates from HPX upon acidification as would occur in the maturing endosome (Smith et al., 2009). Therefore, heparin binding by apo-HPX at acidic pH *in vitro* likely represents an interaction between apo-HPX and a membrane-associated HSPG *in vivo* rather than an effect on heme binding to HPX because absorbance spectroscopy of ligand binding to hemopexin showed that heme competed effectively with heparin (80-fold molar excess) for HPX binding (Figure 8D). Overall, these data support a model whereby heme–HPX could potentially interact with a plasma membrane HSPG, possibly by binding ligands and then “presenting” them, as suggested for LRP1, for endocytosis (Kanekiyo et al., 2011). Importantly, in this model an HSPG in the recycling of apo-HPX by TfRs to the cell surface, similar to their proposed function in apo-Tf recycling. HPX receptors function in heme–HPX endocytosis and in the delivery of heme to specific cells. Furthermore, certain interactions with HSPGs and HPX receptors may aid in determining the cell surface specificity of HPX binding.

## 4 Logical outcomes and future developments

Recycling of HPX was calculated to account for the clearance of a heme load by plasma HPX in humans (Drabkin, 1971), *in vivo* in rats (Smith and Morgan, 1978), in HepG2 cells (Smith and Hunt, 1990), and, as mentioned, in HL-60 cells. The intracellular route of the heme from heme–HPX requires clarification even after the identification of the scavenger receptor LRP1 as a high-affinity heme–HPX receptor. This is because LRP1 targets HPX for lysosomal degradation (Hvidberg et al., 2005), presumably lowering plasma HPX levels in contrast to an HPX recycling receptor system such as TfRs. Ligand affinity isolations here and ICC studies revealed that heme complexes of both human and rabbit HPX bind human TfR1 from HL-60 extracts and from HepG2 and primary human hepatocyte extracts, providing evidence that this binding is specific and physiologically relevant. Heme–HPX recognition by TfR1 provides an ideal mechanism for recycling apo-HPX (Drabkin, 1971), helping maintain HPX plasma levels.

Furthermore, we showed that hemopexin links heme and iron metabolism in various ways (Smith and Ledford, 1988). From a biological standpoint, TfR1 by also binding heme–HPX acts as a scavenger receptor recognizing an array of structurally diverse endogenous proteins, some with key roles in maintaining the cell and whole-body homeostasis of both heme and its iron; thus, it changes our viewpoint of the established mechanisms whereby iron homeostasis is maintained.

Consequently, there are several clinical implications for heme–HPX as an additional ligand for TfR1. The TfR1’s primary role is generally considered to be maintaining cell iron homeostasis. HPX plays key roles in cell iron homeostasis, linking heme and iron metabolism (Davies et al., 1979; Smith and Ledford, 1988), which are not always acknowledged (*e.g.*, growing cells salvage both iron and heme iron for their survival). Cell growth was the same when models of mouse hepatocytes were cultured in defined EMEM (with insulin and selenium) with heme–HPX as the sole source of iron as when iron was provided by diferric–Tf (Smith and Ledford, 1988). Thus, the heme iron released intracellularly from heme transported by HPX enters the same low molecular-mass intracellular pool as does iron from iron-Tf and is utilized for DNA synthesis by ribonucleotide reductase for cell growth.

Therefore, in addition to its role in iron metabolism, TfR1 should be termed a scavenger receptor, which recognizes a “large repertoire of ligands” and generally removes non-native or harmful substances (PrabhuDas et al., 2017). TfR1s bind many molecules, most of which are needed for their role in cellular iron homeostasis, but they also bind additional proteins and several viruses. Not only does the TfR1 bind two molecules of diferric–transferrin, but it also recruits and binds two other proteins that play key roles in maintaining systemic, *i.e.*, whole-body, iron status (Testi et al., 2019). For example, the hereditary hemochromatosis factor protein is a second ligand that binds to TfR1 on a site that overlaps with that of holo-Tf on the helical and protease-like domains of the receptor. This competition decreases the affinity of iron–Tf complexes and, consequently, decreases intracellular iron levels. Another ligand is H-ferritin (which contains iron), secreted by macrophages and hepatocytes. TfR1 also binds to surface proteins on several viruses (Testi et al., 2019), to a protein needed for infection by the malaria parasite *Plasmodium vivax* (Testi et al., 2019), and, more recently, to the severe acute respiratory syndrome coronavirus 2 (SARS-CoV-2) (Liao et al., 2024). Finally, in the context of heme clearance, TfR1 was identified as a surface receptor for heme bound to human serum albumin on an immortalized line of human T lymphocyte cells (Brell et al., 2020). Given the variety of structurally diverse ligands for TfR1 that now includes heme–HPX, TfR1’s classification as a scavenger receptor seems apt. Given this large number of protein ligands for TfR1, many of which play key roles in iron metabolism, together with the fact that HPX links cellular and whole-body heme biology with cell iron homeostasis, our findings that TfR1 is an endocytotic receptor for heme–HPX are completely consistent with the known cell biology of HPX. Furthermore, the binding of heme–HPX and heme–human serum albumin to TfRs provides additional evidence for their important role in heme homeostasis, in addition to their key role in iron metabolism at the cell and whole-body levels.

Nevertheless, our observations do not preclude the existence of an HPXR that is specific for heme–HPX, *i.e.*, binds only heme–HPX complexes at the cell surface. Of particular interest is our finding that heme–HPX binds to the CD 200 receptor 1 protein from HL-60 cell extracts. HPX is known to modulate the immune system, and this receptor interacts with a cell surface and highly glycosylated protein, CD200, to reduce myeloid activity in inflammation and may block cancer cell activities.

Primary human hepatocytes are routinely used for short-term studies of liver function, especially in drug metabolism and liver biology research, in part because members of the cytochrome P-450 system and their drug-metabolizing activity are active in these cells. These proteins, as their name implies, are heme-proteins. Furthermore, HMOX1 levels and heme-requiring transcriptional regulation by heme–HPX improved after a few days of “recovery” of these human primary cells after there initial plating.

The affinity-isolated heme–HPX binding proteins from these primary liver cells and HepG2 cells included both TfR1 and LRP1, confirming and extending the ICC data. The ICC data showing co-localization of TfR2 with HPX have to be considered preliminary because TfR2 was not detected in the isolates from the HPX-charged affinity resins or from the holo-human Tf-Affi-Gel 15. However, TfR2 levels may have been low in the isolates because it was not detected using bovine holo-Tf, a ligand known to preferentially bind TfR but not TfR1 (Kawabata et al., 2004), in the affinity isolations of HepG2 whole-cell extracts (data not shown). High body iron downregulates TfR2, so low levels may reflect the high iron status of the liver donors. Moreover, human hepatocytes are expected to express low levels of TfRs, unlike rapidly dividing cells in the transformed cell lines.

Many of the biological processes in which the HPX interactome proteins are involved are consistent between both the HepG2 cells and primary human liver cells. These include receptor-mediated endocytosis, iron/metal ion transport, and cell homeostasis. However, there are also important differences that are clinically relevant. In the human liver interactome, there is greater involvement of the proteins involved in lipid metabolism and various components involved in immune response activation and receptor-mediated endocytosis. Additional proteins are linked with plasma lipid metabolism, brain and CNS endothelial cell maintenance, and blood pressure regulation and tissue remodeling (kallikrein). The PANTHER GO-Slim analyses revealed an association of HPX with the biological processes that fall into the following groups: hemostasis, regulation of response to wounding, activation of immune response, complement activation, coagulation, regulation of metal iron ion transport, and regulation of body fluid levels (*i.e*., kidney function).

Interestingly, there are parallels between iron uptake from holo-Tf and heme uptake from heme–HPX, both described in terms of two processes. For the Tf system using human melanoma cells, these were termed specific and “non-specific”; both required TfRs, and both iron uptake processes required Tf (Richardson and Baker, 1992). For the HPX system in freshly isolated rat hepatocytes, these were termed specific and “selective” (Smith and Morgan, 1981), via HPX receptors, and both processes required HPX. Furthermore, these specific and selective HPX uptake systems were distinguished based on their different responses to metabolic inhibitors, which inhibited specific binding and heme uptake, while the selective process required metabolic energy but was not saturable. Both processes also occur *in vivo* (Smith and Morgan, 1981). This aspect of both the transferrin and hemopexin systems requires further characterization.

Another overlooked feature of the heme–HPX cell interaction is the activation of signaling cascades, including the N-terminal cJun-kinase (JNK) pathway (Eskew et al., 1999). Signaling may be related to a role for the superfamily of HSPGs. These are integral cell-surface membrane proteins that present ligands, in part via receptors in the extracellular matrix, and activate signaling cascades through protein assemblies on their cytosolic domains. Intracellular signaling cascades are activated by surface binding of heparin and/or HSPGs together with other molecules, including integrins. The mitogen-activated protein kinase (MAPK) pathway, originally termed extracellular receptor kinases (ERKs), includes the Ras/Raf/MEK/ERK pathway.

Furthermore, with respect to apo-HPX recycling in the context of HPX binding to a surface receptor, we consider that heparin binding to apo-HPX, but not heme–HPX, models an interaction between heme–HPX with an HPX receptor-associated surface HSPG, as suggested for holo-Tf and TfR1 (Regoeczi et al., 1994). Such binding likely helps recycle apo-HPX back to the cell surface after the release of heme as the pH decreases in maturing endosomes.

It is not well recognized that plasma heme levels regulate the turnover of HPX (*i.e.*, its rate of synthesis and rate of degradation in hepatocytes). This was first shown in rhesus monkeys (Foidart et al., 1982) and supported by data from patients with hemolytic anemias, neuromuscular diseases, and even in the porphyrias with defective heme synthesis (Foidart et al., 1983). Low levels of heme increase plasma HPX by increasing hepatic synthesis without changing catabolism. High plasma heme levels decrease plasma HPX by increasing catabolism, while medium plasma heme levels increase both synthesis and catabolism; thus, plasma HPX levels are essentially unchanged. However, based on the cell biology of LRP1, LRP1 targets HPX to lysosomes, decreasing hepatic HPX plasma levels in the absence of stimuli to increase HPX synthesis and contrasting with apo-HPX recycling after endocytosis.

Importantly, the data presented here endorse the clinical relevance of the HPX system in several ways. In this context, there are significant differences in the response of the HPX system in human populations and experimental animals, *e.g.*, mice, especially under conditions of acute inflammation where heme is involved (Lin et al., 2015). Therefore, to improve patient care with HPX, there is a need to expand and consolidate knowledge of the processes and molecules that affect plasma HPX levels in human health and disease. This is relevant because HPX is being increasingly investigated as a plasma diagnostic marker, for example, in non-hemolytic, inflammatory states (Larsen et al., 2010; Janz et al., 2013; Jung et al., 2015; Pukajlo-Marczyk and Zwolinska, 2021; Winter et al., 2021) and as a parameter for assessing the severity of hemolytic conditions. Changes in plasma haptoglobin (Hp) levels are difficult to assess because they are affected by multiple factors: decreased response to needed blood transfusions, potentially functional differences among Hp proteoforms, and the fact that Hp is an acute-phase reactant in humans (Gupta et al., 2011; Naryzhny and Legina, 2021). Thus, plasma HPX levels have potential as a useful and reliable diagnostic.

HPX is clearly needed to maintain hemostasis, and this is further supported by the “interactome” data here. Thus, factors and processes that affect HPX plasma levels require definition, not simply to better understand the HPX system but, importantly, to optimize the assessment of changes in plasma HPX levels over time, thereby improving the use of HPX as a diagnostic marker for both humans and animals (Saril et al., 2022). During microgravity, hemolysis develops in astronauts, leading to anemia termed “space anemia” (Trudel et al., 2022); thus, plasma HPX levels will help assess not only the extent of anemia with other clinical parameters that include immune suppression (Wang et al., 2015) but also the restoration of normal hematopoiesis in astronauts and, potentially, space tourists upon their return to earth. The extent of such anemia and recovery from it are related to the duration of time spent in microgravity. Space flights are becoming longer, or as we have observed recently, there are unexpected delays in returning to Earth (astronauts Sunita Williams and Butch Wilmore spent 286 consecutive days in the space station), making such research increasingly necessary and relevant.

HPX is considered part of the innate immune response because of its ability to prevent activation of Toll-like receptor 4 by heme (Belcher et al., 2014) and due to its key role in nutritional immunity (Sakamoto et al., 2017). TfR1–heme–HPX binding has important implications for the interaction of heme–HPX with cells of the human immune system, *e.g.*, human polymorphonuclear cells that function in innate immunity. Finally, interactions between TfRs and heme–HPX provide a key to expanding our understanding of the regulation of the complex relationships between systemic heme and iron metabolism and their clinical relevance and importance; however, many details remain to be fully analyzed and understood.

Overall, the research in this study provides novel information on the intracellular path for HPX after endocytosis into models of human immune system cells and primary human hepatocytes. The ICC studies presented lay a foundation for future research on the cell biology of HPX. Affinity isolations have identified key human proteins and biological processes in which the HPX system is involved. Furthermore, the data support that both the HPX and Tf systems are closely linked to lipid metabolism, immune system activation and function, and their respective roles in heme and iron homeostasis. Thus, a broader understanding of heme-related parameters, hepatic heme, and HPX metabolism and how they affect plasma HPX levels will help improve disease prognosis and therapeutics.

## Data Availability

The datasets presented in this study can be found in online repositories. The names of the repository/repositories and accession number(s) can be found in the article/Supplementary Material.

## References

[B1] Ajayi A. S. Gerkins C. Fragoso G. Calve A. Santos M. M. (2025). Hemopexin and HO-1 induction during acute colitis in mice is dependent on interleukin-22. Front. Immunol. 16, 1614466. 10.3389/fimmu.2025.1614466 40791589 PMC12336042

[B2] Alam J. Smith A. (1989). Receptor-mediated transport of heme by hemopexin regulates gene expression in mammalian cells. J. Biol. Chem. 264, 17637–17640. 10.1016/s0021-9258(19)84616-1 2553689

[B105] Alam J. Smith A. (1992). Heme-hemopexin-mediated induction of metallothionein gene expression. J. Biol. Chem. 267 (23), 16379–16384. 1644822

[B3] Alam J. Stewart D. Touchard C. Boinapally S. Choi A. M. Cook J. L. (1999). Nrf2, a Cap’n’Collar transcription factor, regulates induction of the heme oxygenase-1 gene. J. Biol. Chem. 274 (37), 26071–26078. 10.1074/jbc.274.37.26071 10473555

[B4] Ardisasmita A. I. Schene I. F. Joore I. P. Kok G. Hendriks D. Artegiani B. (2022). A comprehensive transcriptomic comparison of hepatocyte model systems improves selection of models for experimental use. Commun. Biol. 5 (1), 1094. 10.1038/s42003-022-04046-9 36241695 PMC9568534

[B5] Ashouri R. Fangman M. Burris A. Ezenwa M. O. Wilkie D. J. Dore S. (2021). Critical role of hemopexin mediated cytoprotection in the pathophysiology of sickle cell disease. Int. J. Mol. Sci. 22 (12), 6408. 10.3390/ijms22126408 34203861 PMC8232622

[B6] Ashraf A. A. Dani M. So P. W. (2020). Low cerebrospinal fluid levels of hemopexin are associated with increased alzheimer's pathology, hippocampal hypometabolism, and cognitive decline. Front. Mol. Biosci. 7, 590979. 10.3389/fmolb.2020.590979 33392254 PMC7775585

[B7] Belcher J. D. Chen C. Nguyen J. Milbauer L. Abdulla F. Alayash A. I. (2014). Heme triggers TLR4 signaling leading to endothelial cell activation and vaso-occlusion in murine sickle cell disease. Blood 123123 (3), 377–390. 10.1182/blood-2013-04-495887 24277079 PMC3894494

[B8] Birnie G. D. (1988). The HL60 cell line: a model system for studying human myeloid cell differentiation. Br. J. Cancer Suppl. 9, 41–45. 3076064 PMC2149104

[B9] Bjornevik K. Cortese M. Healy B. C. Kuhle J. Mina M. J. Leng Y. (2022). Longitudinal analysis reveals high prevalence of Epstein-Barr virus associated with multiple sclerosis. Science 375 (6578), 296–301. 10.1126/science.abj8222 35025605

[B10] Borza D. B. Morgan W. T. (1998). Histidine-proline-rich glycoprotein as a plasma pH sensor. Modulation of its interaction with glycosaminoglycans by ph and metals. J. Biol. Chem. 273 (10), 5493–5499. 10.1074/jbc.273.10.5493 9488672

[B11] Brell J. Berg V. Modak M. Puck A. Seyer-Jiresch M. al e. (2020). Transferrin receptor 1 is a cellular receptor for human heme-albumin. Commun. Biol. 3, 621. 10.1038/s42003-020-01294-5 33110194 PMC7591885

[B12] Casey J. L. Hentze M. W. Koeller D. M. Caughman S. W. Rouault T. A. Klausner R. D. (1988). Iron-responsive elements: regulatory RNA sequences that control mRNA levels and translation. Science 240, 924–928. 10.1126/science.2452485 2452485

[B13] Collins J. M. Foster K. A. (1983). Differentiation of promyelocytic (HL-60) cells into mature granulocytes: mitochondrial-specific rhodamine 123 fluorescence. J. Cell Biol. 96 (1), 94–99. 10.1083/jcb.96.1.94 6572192 PMC2112239

[B14] Collins S. J. Gallo R. C. Gallagher R. E. (1977). Continuous growth and differentiation of human myeloid leukaemic cells in suspension culture. Nature 270 (5635), 347–349. 10.1038/270347a0 271272

[B15] Conner S. D. Schmid S. L. (2003). Regulated portals of entry into the cell. Nature 422 (6927), 37–44. 10.1038/nature01451 12621426

[B16] Correia M. A. Sinclair P. R. De Matteis F. (2011). Cytochrome P450 regulation: the interplay between its heme and apoprotein moieties in synthesis, assembly, repair, and disposal. Drug Metab. Rev. 43 (1), 1–26. 10.3109/03602532.2010.515222 20860521 PMC3034403

[B17] Davies D. M. Smith A. Muller-Eberhard U. Morgan W. T. (1979). Hepatic subcellular metabolism of heme from heme-hemopexin: incorporation of iron into ferritin. Biochem. Biophys. Res. Commun. 91 (4), 1504–1511. 10.1016/0006-291x(79)91235-x 526319

[B18] Drabkin D. L. (1971). Heme binding and transport--a spectrophotometric study of plasma glycoglobulin hemochromogens. Proc. Natl. Acad. Sci. U. S. A. 68 (3), 609–613. 10.1073/pnas.68.3.609 4100308 PMC389000

[B19] Engelberg H. (1961). Plasma heparin levels in normal man. Circulation 23, 578–581. 10.1161/01.cir.23.4.578 13696820

[B20] Enns C. A. Sussman H. H. (1981). Physical characterization of the transferrin receptor in human placentae. J. Biol. Chem. 256 (19), 9820–9823. 10.1016/s0021-9258(19)68700-4 6268632

[B21] Eskew J. D. Vanacore R. M. Sung L. Morales P. J. Smith A. (1999). Cellular protection mechanisms against extracellular heme: heme-hemopexin, but not free heme, activates the N-terminal c-Jun kinase. J. Biol. Chem. 274 (2), 638–648. 10.1074/jbc.274.2.638 9872997

[B22] Fagoonee S. Di Cunto F. Vozzi D. Volinia S. Pellegrino M. Gasparini P. (2006). Microarray and large-scale in silico--based identification of genes functionally related to haptoglobin and/or hemopexin. DNA Cell Biol. 25 (6), 323–330. 10.1089/dna.2006.25.323 16792502

[B23] Foidart M. Eiseman J. Engel W. K. Adornato B. T. Liem H. H. Muller-Eberhard U. (1982). Effect of heme administration on hemopexin metabolism in the rhesus monkey. J. Lab. Clin. Med. 100 (3), 451–460. 7108353

[B24] Foidart M. Liem H. H. Adornato B. T. Engel W. K. Muller-Eberhard U. (1983). Hemopexin metabolism in patients with altered serum levels. J. Lab. Clin. Med. 102 (5), 838–846. 6631174

[B25] Graffmann N. Scherer B. Adjaye J. (2022). *In vitro* differentiation of pluripotent stem cells into hepatocyte like cells - basic principles and current progress. Stem Cell Res. 61, 102763. 10.1016/j.scr.2022.102763 35395623

[B26] Grant B. D. Donaldson J. G. (2009). Pathways and mechanisms of endocytic recycling. Nat. Rev. Mol. Cell Biol. 10 (9), 597–608. 10.1038/nrm2755 19696797 PMC3038567

[B27] Gupta S. Ahern K. Nakhl F. Forte F. (2011). Clinical usefulness of haptoglobin levels to evaluate hemolysis in recently transfused patients. Adv. Hematol. 2011, 389854. 10.1155/2011/389854 21860624 PMC3153881

[B28] Hada H. Shiraki T. Watanabe-Matsui M. Igarashi K. (2014). Hemopexin-dependent heme uptake *via* endocytosis regulates the Bach1 transcription repressor and heme oxygenase gene activation. Biochim. Biophys. Acta 1840 (7), 2351–2360. 10.1016/j.bbagen.2014.02.029 24613679

[B29] Hahl P. Hunt R. Bjes E. S. Skaff A. Keightley J. A. Smith A. (2017). Identification of oxidative modifications of hemopexin and their predicted physiological relevance. J. Biol. Chem. 292, 13658–13671. 10.1074/jbc.M117.783951 28596380 PMC5566522

[B30] Haile D. J. Hentze M. W. Rouault T. A. Harford J. B. Klausner R. D. (1989). Regulation of interaction of the iron-responsive element binding protein with iron-responsive RNA elements. Mol.Cell Biol. 9, 5055–5061. 10.1128/mcb.9.11.5055-5061.1989 2601708 PMC363657

[B31] Harford J. B. Klausner R. D. (1990). Coordinate post-transcriptional regulation of ferritin and transferrin receptor expression: the role of regulated RNA-protein interaction. Enzyme 44, 28–41. 10.1159/000468745 2133655

[B32] Hu W. L. Regoeczi E. (1992). Hepatic heparan sulphate proteoglycan and the recycling of transferrin. Biochem. Cell Biol. 70 (7), 535–538. 10.1139/o92-083 1449722

[B33] Hvidberg V. Maniecki M. B. Jacobsen C. Hojrup P. Moller H. J. Moestrup S. K. (2005). Identification of the receptor scavenging hemopexin-heme complexes. Blood 106 (7), 2572–2579. 10.1182/blood-2005-03-1185 15947085

[B34] Janz D. R. Bastarache J. A. Sills G. Wickersham N. May A. K. Bernard G. R. (2013). Association between haptoglobin, hemopexin and mortality in adults with sepsis. Crit. Care 17 (6), R272. 10.1186/cc13108 24225252 PMC4056258

[B35] Jing S. Q. Trowbridge I. S. (1987). Identification of the intermolecular disulfide bonds of the human transferrin receptor and its lipid-attachment site. EMBO J. 6 (2), 327–331. 10.1002/j.1460-2075.1987.tb04758.x 3582362 PMC553399

[B36] Johnson M. B. Enns C. A. (2004). Diferric transferrin regulates transferrin receptor 2 protein stability. Blood 104 (13), 4287–4293. 10.1182/blood-2004-06-2477 15319290

[B37] Jovic M. Sharma M. Rahajeng J. Caplan S. (2010). The early endosome: a busy sorting station for proteins at the crossroads. Histol. Histopathol. 25 (1), 99–112. 10.14670/HH-25.99 19924646 PMC2810677

[B38] Jung J. Y. Kwak Y. H. Kim K. S. Kwon W. Y. Suh G. J. (2015). Change of hemopexin level is associated with the severity of sepsis in endotoxemic rat model and the outcome of septic patients. J. Crit. Care 30 (3), 525–530. 10.1016/j.jcrc.2014.12.009 25588861

[B39] Kanekiyo T. Zhang J. Liu Q. Liu C. C. Zhang L. Bu G. (2011). Heparan sulphate proteoglycan and the low-density lipoprotein receptor-related protein 1 constitute major pathways for neuronal amyloid-beta uptake. J. Neurosci. 31 (5), 1644–1651. 10.1523/JNEUROSCI.5491-10.2011 21289173 PMC3272839

[B40] Kawabata H. Tong X. Kawanami T. Wano Y. Hirose Y. Sugai S. (2004). Analyses for binding of the transferrin family of proteins to the transferrin receptor 2. Br. J. Haematol. 127 (4), 464–473. 10.1111/j.1365-2141.2004.05224.x 15521925

[B41] Kleven M. D. Jue S. Enns C. A. (2018). Transferrin receptors TfR1 and TfR2 bind transferrin through differing mechanisms. Biochemistry 57 (9), 1552–1559. 10.1021/acs.biochem.8b00006 29388418 PMC6038944

[B42] Kounnas M. Z. Argraves W. S. Strickland D. K. (1992a). The 39-kDa receptor-associated protein interacts with two members of the low density lipoprotein receptor family, alpha 2-macroglobulin receptor and glycoprotein 330. J. Biol. Chem. 267 (29), 21162–21166. 10.1016/s0021-9258(19)36811-5 1400426

[B43] Kounnas M. Z. Morris R. E. Thompson M. R. FitzGerald D. J. Strickland D. K. Saelinger C. B. (1992b). The alpha 2-macroglobulin receptor/low density lipoprotein receptor-related protein binds and internalizes *Pseudomonas exotoxin* A. J. Biol. Chem. 267 (18), 12420–12423. 10.1016/s0021-9258(18)42291-0 1618748

[B44] Laatsch A. Panteli M. Sornsakrin M. Hoffzimmer B. Grewal T. Heeren J. (2012). Low density lipoprotein receptor-related protein 1 dependent endosomal trapping and recycling of apolipoprotein E. PLoS One 7 (1), e29385. 10.1371/journal.pone.0029385 22238606 PMC3251589

[B45] Larsen R. Gozzelino R. Jeney V. Tokaji L. Bozza F. A. Japiassu A. M. (2010). A central role for free heme in the pathogenesis of severe sepsis. Sci. Transl. Med. 2 (51), 51ra71. 10.1126/scitranslmed.3001118 20881280

[B107] Li R. C. Saleem S. Zhen G. Cao W. Zhuang H. Lee J. (2009). Heme-hemopexin complex attenuates neuronal cell death and stroke damage. J. Cereb. Blood Flow Metab. 29 (5), 953–964. 19277051 10.1038/jcbfm.2009.19 PMC6015738

[B46] Li T. Adams J. Zhu P. Zhang T. Tu F. Gravitte A. (2025). The role of heme in sepsis induced Kupffer cell PANoptosis and senescence. Cell Death Dis. 16 (1), 284. 10.1038/s41419-025-07637-6 40221420 PMC11993645

[B47] Liao Z. Wang C. Tang X. Yang M. Duan Z. Liu L. (2024). Human transferrin receptor can mediate SARS-CoV-2 infection. Proc. Natl. Acad. Sci. U. S. A. 121 (10), e2317026121. 10.1073/pnas.2317026121 38408250 PMC10927525

[B48] Liem H. H. (1976). Catabolism of homologous and heterologous hemopexin in the rat and uptake of hemopexin by isolated perfused rat liver. Ann. Clin. Res. 17, 233–238. 1008495

[B49] Lijnen H. R. Hoylaerts M. Collen D. (1983). Heparin binding properties of human histidine-rich glycoprotein mechanism and role in the neutralization of heparin in plasma. J. Biol. Chem. 258, 3803–3808. 10.1016/s0021-9258(18)32737-6 6833231

[B50] Lin T. Maita D. Thundivalappil S. R. Riley F. E. Hambsch J. Van Marter L. J. (2015). Hemopexin in severe inflammation and infection: mouse models and human diseases. Crit. Care 19, 166. 10.1186/s13054-015-0885-x 25888135 PMC4424824

[B108] Majuri R. Grasbeck R. (1986). Isolation of the haemopexin-haem receptor from pig liver cells. FEBS Lett. 199, 80–84. 10.1016/0014-5793(86)81227-3 3007216

[B51] Marakasova E. Olivares P. Karnaukhova E. Chun H. Hernandez N. E. Kurasawa J. H. (2021). Molecular chaperone RAP interacts with LRP1 in a dynamic bivalent mode and enhances folding of ligand-binding regions of other LDLR family receptors. J. Biol. Chem. 297 (1), 100842. 10.1016/j.jbc.2021.100842 34058195 PMC8239462

[B52] Mayle K. M. Le A. M. Kamei D. T. (2012). The intracellular trafficking pathway of transferrin. Biochim. Biophys. Acta 1820 (3), 264–281. 10.1016/j.bbagen.2011.09.009 21968002 PMC3288267

[B53] Mi H. Muruganujan A. Huang X. Ebert D. Mills C. Guo X. (2019). Protocol update for large-scale genome and gene function analysis with the PANTHER classification system (v.14.0). Nat. Protoc. 14 (3), 703–721. 10.1038/s41596-019-0128-8 30804569 PMC6519457

[B54] Mihara K. Nakayama T. Saitoh H. (2015). A convenient technique to fix suspension cells on a coverslip for microscopy. Curr. Protoc. Cell Biol. 68 (4), 4.30.1–4.30.10. 10.1002/0471143030.cb0430s68 26331985

[B55] Miller Y. I. Smith A. Morgan W. T. Shaklai N. (1996). Role of hemopexin in protection of low-density lipoprotein against hemoglobin-induced oxidation. Biochemistry 35 (40), 13112–13117. 10.1021/bi960737u 8855948

[B56] Mojzikova R. Koralkova P. Holub D. Zidova Z. Pospisilova D. Cermak J. (2014). Iron status in patients with pyruvate kinase deficiency: neonatal hyperferritinaemia associated with a novel frameshift deletion in the PKLR gene (p.Arg518fs), and low hepcidin to ferritin ratios. Br. J. Haematol. 165 (4), 556–563. 10.1111/bjh.12779 24533562

[B104] Montecinos L. Eskew J. D. Smith A. (2019). What is next in this “age” of heme-driven pathology and protection by hemopexin? an update and links with iron. Pharmaceuticals (Basel) 12 (4) 10.3390/ph12040144 31554244 PMC6958331

[B109] Morgan W. T. Muster P. Tatum F. Kao S. M. Alam J. Smith A. (1993). Identification of the histidine residues of hemopexin that coordinate with heme-iron and of a receptor-binding region. J. Biol. Chem. 268 (9), 6256–6262. 7681064

[B57] Mucci D. Forristal J. Strickland D. Morris R. Fitzgerald D. Saelinger C. B. (1995). Level of receptor-associated protein moderates cellular susceptibility to pseudomonas exotoxin A. Infect. Immun. 63 (8), 2912–2918. 10.1128/iai.63.8.2912-2918.1995 7622212 PMC173396

[B58] Muckenthaler M. U. Galy B. Hentze M. W. (2008). Systemic iron homeostasis and the iron-responsive element/iron-regulatory protein (IRE/IRP) regulatory network. Annu. Rev. Nutr. 28, 197–213. 10.1146/annurev.nutr.28.061807.155521 18489257

[B110] Muller-Eberhard U. Javid J. Liem H. H. Hanstein A. Hanna M. (1968). Plasma concentrations of hemopexin, haptoglobin and heme in patients with various hemolytic diseases. Blood 32 (5), 811–815. 5687939

[B59] Naryzhny S. N. Legina O. K. (2021). Haptoglobin as a biomarker. Biomed. Khim 67 (2), 105–118. 10.18097/PBMC20216702105 33860767

[B60] Ori A. Free P. Courty J. Wilkinson M. C. Fernig D. G. (2009). Identification of heparin-binding sites in proteins by selective labeling. Mol. Cell Proteomics 8 (10), 2256–2265. 10.1074/mcp.M900031-MCP200 19567366 PMC2758754

[B61] Paoli M. Anderson B. F. Baker H. M. Morgan W. T. Smith A. Baker E. N. (1999). Crystal structure of hemopexin reveals a novel high-affinity heme site formed between two beta-propeller domains. Nat. Struct. Biol. 6 (10), 926–931. 10.1038/13294 10504726

[B62] Poillerat V. Gentinetta T. Leon J. Wassmer A. Edler M. Torset C. (2020). Hemopexin as an inhibitor of hemolysis-induced complement activation. Front. Immunol. 11, 1684. 10.3389/fimmu.2020.01684 32849588 PMC7412979

[B63] PrabhuDas M. R. Baldwin C. L. Bollyky P. L. Bowdish D. M. E. Drickamer K. Febbraio M. (2017). A consensus definitive classification of scavenger receptors and their roles in health and disease. J. Immunol. 198 (10), 3775–3789. 10.4049/jimmunol.1700373 28483986 PMC5671342

[B64] Pukajlo-Marczyk A. Zwolinska D. (2021). Involvement of hemopexin in the pathogenesis of proteinuria in children with idiopathic nephrotic syndrome. J. Clin. Med. 10 (14), 3160. 10.3390/jcm10143160 34300326 PMC8303445

[B65] Regoeczi E. Chindemi P. A. Hu W. L. (1994). Interaction of transferrin and its iron-binding fragments with heparin. Biochem. J. 299 (Pt 3), 819–823. 10.1042/bj2990819 8192672 PMC1138094

[B66] Richardson D. Baker E. (1992). Two mechanisms of iron uptake from transferrin by melanoma cells. The effect of desferrioxamine and ferric ammonium citrate. J. Biol. Chem. 267 (20), 13972–13979. 10.1016/s0021-9258(19)49665-8 1629195

[B67] Rish K. R. Swartzlander R. Sadikot T. N. Berridge M. V. Smith A. (2007). Interaction of heme and heme–hemopexin with an extracellular oxidant system used to measure cell growth-associated plasma membrane electron transport. Biochem. Biophys. Acta Bioenerg. 1767 (9), 1107–1117. 10.1016/j.bbabio.2007.06.003 17643387

[B68] Rouault T. A. Tang C. K. Kaptain S. Burgess W. H. Haile D. J. Samaniego F. (1990). Cloning of the cDNA encoding an RNA regulatory protein-- the human iron-responsive element-binding protein. Proc. Natl. Acad. Sci. U. S. A. 87, 7958–7962. 10.1073/pnas.87.20.7958 2172968 PMC54871

[B69] Sakamoto K. Kim Y. G. Hara H. Kamada N. Caballero-Flores G. Tolosano E. (2017). IL-22 controls iron-dependent nutritional immunity against systemic bacterial infections. Sci. Immunol. 2 (8), eaai8371. 10.1126/sciimmunol.aai8371 28286877 PMC5345941

[B70] Salvioli S. Dobrucki J. Moretti L. Troiano L. Fernandez M. G. Pinti M. (2000). Mitochondrial heterogeneity during staurosporine-induced apoptosis in HL60 cells: analysis at the single cell and single organelle level. Cytometry 40 (3), 189–197. 10.1002/1097-0320(20000701)40:3<189::aid-cyto3>3.0.co;2-6 10878561

[B71] Saril A. Kocaturk M. Shimada K. Uemura A. Akgun E. Levent P. (2022). Serum proteomic changes in dogs with different stages of chronic heart failure. Anim. (Basel) 12 (4), 490. 10.3390/ani12040490 35203200 PMC8868296

[B106] Smith A. Eskew J. D. Borza C. M. Pendrak M. Hunt R. C. (1997). Role of heme-hemopexin in human T-lymphocyte proliferation. Exp. Cell Res. 232 (2), 246–254. 10.1006/excr.1997.3526 9168799

[B72] Smith A. (2011). “Mechanisms of cytoprotection by hemopexin,” in Handbook of porphyrin science. Biochemistry of tetrapyrroles. Editors Kadish K. M. Smith K. M. Guilard R. (Singapore: World Scientific Publishing Co. Pte. Ltd.), 217–356.

[B73] Smith A. Hunt R. C. (1990). Hemopexin joins transferrin as representative members of a distinct class of receptor-mediated endocytic transport systems. Eur. J. Cell Biol. 53, 234–245. 1964416

[B74] Smith A. Ledford B. E. (1988). Expression of the haemopexin-transport system in cultured mouse hepatoma cells. Links between haemopexin and iron metabolism. Biochem. J. 256, 941–950. 10.1042/bj2560941 2852010 PMC1135507

[B75] Smith A. McCulloh R. J. (2016). Mechanisms of haem toxicity in haemolysis and protection by the haem-binding protein, haemopexin. ISBT Sci. Ser. Congr. Rev. PL1-01 Ed. P. van der Meer 12 (1), 119–133. 10.1111/voxs.12340

[B76] Smith A. Morgan W. T. (1978). Transport of heme by hemopexin to the liver: evidence for receptor-mediated uptake. Biochem. Biophys. Res. Commun. 84, 151–157. 10.1016/0006-291x(78)90276-0 728123

[B77] Smith A. Morgan W. T. (1979). Haem transport to the liver by haemopexin. Receptor-mediated uptake with recycling of the protein. Biochem. J. 182 (1), 47–54. 10.1042/bj1820047 496916 PMC1161233

[B78] Smith A. Morgan W. T. (1981). Hemopexin-mediated transport of heme into isolated rat hepatocytes. J. Biol. Chem. 256, 10902–10909. 10.1016/s0021-9258(19)68530-3 7287740

[B79] Smith A. Morgan W. T. (1985). Hemopexin-mediated heme transport to the liver. Evidence for a heme-binding protein in liver plasma membranes. J. Biol. Chem. 260, 8325–8329. 10.1016/s0021-9258(17)39475-9 3891754

[B80] Smith A. Alam J. Escriba P. V. Morgan W. T. (1993). Regulation of heme oxygenase and metallothionein gene expression by the heme analogs, cobalt-and tin-protoporphyrin. J. Biol. Chem. 268 (10), 7365–7371. 10.1016/s0021-9258(18)53184-7 8463269

[B81] Smith A. Rish K. R. Lovelace R. Hackney J. F. Helston R. M. (2009). Role for copper in the cellular and regulatory effects of heme-hemopexin. Biometals 22, 421–437. 10.1007/s10534-008-9178-z 19039664

[B82] Sun J. Hoshino H. Takaku K. Nakajima O. Muto A. Suzuki H. (2002). Hemoprotein Bach1 regulates enhancer availability of heme oxygenase-1 gene. Embo J. 21 (19), 5216–5224. 10.1093/emboj/cdf516 12356737 PMC129038

[B83] Sung L. Morales P. Shibata M. Shipulina N. Smith A. (2000). “Defenses against extracellular heme-mediated oxidative damage: use of iron and copper chelators to investigate the role of redox active iron, copper and heme in the hemopexin heme transport system,” in Iron chelators: new development strategies. Editors Badman D. G. Bergeron R. J. Brittenham G. M. (Sarotoga, Florida: Saratoga Publishing Group), 67–86.

[B84] Suriawinata E. Mehta K. J. (2023). Iron and iron-related proteins in COVID-19. Clin. Exp. Med. 23 (4), 969–991. 10.1007/s10238-022-00851-y 35849261 PMC9289930

[B85] Taetle R. Rhyner K. Castagnola J. To D. Mendelsohn J. (1985). Role of transferrin, Fe, and transferrin receptors in myeloid leukemia cell growth. Studies with an antitransferrin receptor monoclonal antibody. J. Clin. Invest. 75 (3), 1061–1067. 10.1172/JCI111768 2984253 PMC423664

[B86] Takahashi N. Takahashi Y. Putnam F. W. (1985). Complete amino acid sequence of human hemopexin, the heme-binding protein of serum. Proc. Natl. Acad. Sci. U. S. A. 82, 73–77. 10.1073/pnas.82.1.73 3855550 PMC396973

[B87] Takahashi S. Kubo K. Waguri S. Yabashi A. Shin H. W. Katoh Y. (2012). Rab11 regulates exocytosis of recycling vesicles at the plasma membrane. J. Cell Sci. 125 (Pt 17), 4049–4057. 10.1242/jcs.102913 22685325

[B88] Taketani S. Kohno H. Naitoh Y. Tokunaga R. (1987a). Isolation of the hemopexin receptor from human placenta. J. Biol. Chem. 262, 8668–8671. 10.1016/s0021-9258(18)47465-0 3036819

[B89] Taketani S. Kohno H. Tokunaga R. (1987b). Cell surface receptor for hemopexin in human leukemia HL60 cells specific binding, affinity labeling, and fate of heme. J. Biol. Chem. 262, 4639–4643. 10.1016/s0021-9258(18)61241-4 3031028

[B90] Taketani S. Kohno H. Sawamura T. Tokunaga R. (1990). Hemopexin-dependent down-regulation of expression of the human transferrin receptor. J. Biol. Chem. 265 (23), 13981–13985. 10.1016/s0021-9258(18)77445-0 2380200

[B91] Testi C. Boffi A. Montemiglio L. C. (2019). Structural analysis of the transferrin receptor multifaceted ligand(s) interface. Biophys. Chem. 254, 106242. 10.1016/j.bpc.2019.106242 31419721

[B92] Trott O. Olson A. J. (2010). AutoDock vina: improving the speed and accuracy of docking with a new scoring function, efficient optimization, and multithreading. J. Comput. Chem. 31 (2), 455–461. 10.1002/jcc.21334 19499576 PMC3041641

[B93] Trudel G. Shahin N. Ramsay T. Laneuville O. Louati H. (2022). Hemolysis contributes to anemia during long-duration space flight. Nat. Med. 28 (1), 59–62. 10.1038/s41591-021-01637-7 35031790 PMC8799460

[B94] Vallelian F. Buehler P. W. Schaer D. J. (2022). Hemolysis, free hemoglobin toxicity, and scavenger protein therapeutics. Blood 140 (17), 1837–1844. 10.1182/blood.2022015596 35660854 PMC10653008

[B95] Vanacore R. Eskew J. D. Sung L. Davis T. Smith A. (2019). Safe coordinated trafficking of heme and iron with copper maintain cell homeostasis: modules from the hemopexin system. Biometals 32 (3), 355–367. 10.1007/s10534-019-00194-4 31011852

[B96] Vestal D. J. Davis B. H. Enns C. A. (1990). A rapid redistribution of the transferrin receptor to the cell surface of HL-60 cells and K562 cells upon treatment with dimethyl sulfoxide due to slowing of endocytosis. Arch. Biochem. Biophys. 276, 278–284. 10.1016/0003-9861(90)90039-2 2297227

[B97] Vinchi F. Costa da Silva M. Ingoglia G. Petrillo S. Brinkman N. Zuercher A. (2016). Hemopexin therapy reverts heme-induced proinflammatory phenotypic switching of macrophages in a mouse model of sickle cell disease. Blood 127 (4), 473–486. 10.1182/blood-2015-08-663245 26675351 PMC4850229

[B98] Walsh T. G. Li Y. Williams C. M. Aitken E. W. Andrews R. K. Poole A. W. (2021). Loss of the exocyst complex component EXOC3 promotes hemostasis and accelerates arterial thrombosis. Blood Adv. 5, 674–686. 10.1182/bloodadvances.2020002515 33560379 PMC7876885

[B99] Wang C. Li N. Zhang C. Sun S. Gao Y. Long M. (2015). Effects of simulated microgravity on functions of neutrophil-like HL-60 cells. Microgravity Sci. Technol. 27, 515–527. 10.1007/s12217-015-9473-6

[B100] Winter N. A. Gibson P. G. Fricker M. Simpson J. L. Wark P. A. McDonald V. M. (2021). Hemopexin: a novel anti-inflammatory marker for distinguishing COPD from asthma. Allergy Asthma Immunol. Res. 13 (3), 450–467. 10.4168/aair.2021.13.3.450 33733639 PMC7984952

[B111] Wochner R. D. Spilberg I. Iio A. Liem H. H. Muller-Eberhard U. (1974). Hemopexin metabolism in sickle-cell disease, porphyrias and control subjects--effects of heme injection. N. Engl. J. Med. 290 (15), 822–826. 10.1056/NEJM197404112901503 4817836

[B101] Worthen C. A. Enns C. A. (2014). The role of hepatic transferrin receptor 2 in the regulation of iron homeostasis in the body. Front. Pharmacol. 5, 34. 10.3389/fphar.2014.00034 24639653 PMC3944196

[B102] Yeung Y. G. Stanley E. R. (2009). A solution for stripping antibodies from polyvinylidene fluoride immunoblots for multiple reprobing. Anal. Biochem. 389 (1), 89–91. 10.1016/j.ab.2009.03.017 19303392 PMC2679975

[B103] Zeng D. Mizutani K. Qi X. Asada-Utsugi M. Wu B. Kawasaki T. (2025). The association of hemopexin, muscle quality, and sarcopenia in Japanese older adults with cognitive impairment: a cross-sectional study. BMC Geriatr. 25 (1), 332. 10.1186/s12877-025-05977-8 40361004 PMC12070561

