# Supplementary material for: Tracking hemopexin intracellularly and defining hemopexin protein “interactomes” in human immune and liver cell models
Source: Front Physiol. 2025 Nov 20;16:1613917. doi: 10.3389/fphys.2025.1613917 (PMC12690290; doi:10.3389/fphys.2025.1613917)
Supplement: Supplementary file 2 [file Supplementaryfile1.docx]

Supplementary Material

# Supplementary Materials & Methods

*Proteins*: HPX is highly conserved and we have shown that several species of HPX interact with receptors on liver and other cells, including the heme complexes of rabbit and human HPX are functionally equivalent (Morgan et al., 1988). Both proto- and meso-heme complexes of human HPX and rabbit HPX. Extinction coefficients (A.M-1.cm-1) used were 1.1 x 10^5^ at 280 nm for apo-HPX and for heme-HPX 1.2 x 10^5^ at 280 nm and 1.4 x 10^5^ at 405 nm for heme-HPX. Both hemes are known HMOX1 inducers and are similarly bound to HPX via a bis-histidyl coordination (Morgan et al., 1993). However, human HPX has an additional O-linked oligosaccharide on the N-terminal Threonine that the rabbit HPX lacks (Takahashi et al., 1984); nevertheless, HPX is highly conserved at the amino acid level (79%) and the structure of the human HPX protein can be threaded onto the heme-rabbit HPX crystal structure (Paoli et al., 1999). Rabbit HPX is a potential model for certain human O-glycosidase deficiencies that while categorized as “rare diseases” do include muscular dystrophy. *Cell Culture*: HL-60 cells in Iscove’s Modified Dulbecco’s Medium supplemented with 20% FBS and 1% penicillin/ streptomycin as recommended by ATCC (i.e. maintained between 2 x 10^5^ to 1.5 x 10^6^ cells/ml), at 37 °C in 5% CO2. LRP1 negative mouse embryonic fibroblast PEA13 (ATCC RL-2216) cells are homozygous for disruption of the LRP1 gene and were maintained in ATCC-formulated DMEM medium supplemented with 10% FBSand 1% penicillin/streptomycin. PEA 13 cells with the MEF-1 (see ATCC CRL-2214) and PEA 10 (see ATCC CRL-2215) cell lines constitute a genetically defined system to study the endocytosis of ligands by LRP1. Levels of LRP1 in WT CHO cells were compared with LRP1-/-CHO 13-5-1 cells, HL60 and PEA 13 cells. We monitored the small subunit of LRP1 because the large subunit is susceptible to proteolysis in whole cell extracts even in the presence of protease inhibitors (Dr. D. K. Strickland, personal communication).

*Surface biotinylation of HL-60 cells*: Briefly, ~ 40-50 x 10^6^ HL-60 cells were harvested, washed three times with ice cold PBS pH 8. To label the surface proteins the cells were incubated at 4 °C for 1h with the cross-linker EZ-link Sulfo-NHS-biotin (Thermo Fisher Scientific, Lenexa, KS) following company protocols. The reaction was quenched by washing the cells three times with PBS containing 100mM glycine. Then cell extracts were prepared using a HEPES-buffer containing Triton-X100 lysis buffer (50mM HEPES, pH 7.4, 150mM NaCl, 0.5% Triton X-100, containing phosphatase inhibitor cocktail solution 2 , 1X complete protease inhibitor cocktail 1 (Research Product International, Mount Prospect, IL), 2mM phenylmethylsulphonyl fluoride (PMSF) followed by incubation with gentle mixing at 4°C for 1h. Solubilized proteins were recovered in the supernatant after centrifugation (100,000 x g, 30min). Protein concentrations of cell extracts were determined using the BCA assay (Thermo Fisher Scientific, Lenexa, KS).

*Immunocytochemistry (antibodies)*: Primary antibodies to LRP1 (1:50 Abcam, ab-92544, Cambridge, MA), TfR2 (1:50 Santa Cruz Biotechnology, sc-32271, Santa Cruz, CA), Rab 5 (1:100 Abcam, ab-18211, Cambridge, MA), TfR1 (1:100 Santa Cruz Biotechnology, sc-65882, Santa Cruz, CA) and Na+K+ATPase (1:100 Abcam, ab-76020 Cambridge, MA) and AlexaFluor-labeled secondary antibodies Goat anti-Rabbit IgG Alexaflour 546 8µg/ml LRP1, TfR1, Na+K+ATPase , Goat anti-Rabbit IgG Alexafluor 488 5µg/ml Rab5, Goat anti-Mouse IgG Alexaflour 488 2µg/ml & 405 1:25 TfR1 (Thermo Fisher Scientific, Lenexa, KS) TfR2 secondary antibody Mouse IgG BP CFL Alexafluor 555 used at 1:1000 (Santa Cruz Biotechnology, sc-533662, Santa Cruz, CA) First, the specificity of the secondary antibodies for primary targets was determined by ensuring there was no significant fluorescence after incubating fixed cells with secondary antibodies alone using a range of dilutions.

*Contrast Normalization and Gaussian Blur*: The dynamic range of fluorescence signal between the two channels used for co-localization analysis was normalized prior to co-localization analysis. A small gaussian blur of 1 sigma radius was applied to all images to minimize the effects of pixel noise in images where the signal was low. For the down regulation of TfRs, Otsu thresholding was used to create a mask around regions of interest with punctate labeling. Mean pixel values for the entirety of each image were used for data analysis. In some instances, specific regions of interest were hand drawn for single cells or sub-cellular regions and separately analyzed.

*Additions to section on MS analyses of affinity isolated proteins from HL60, HepG2, and human hepatocytes:* FASP Methods – Orbitrap Eclipse (IdeA Proteomics): Protein samples from HepG2 (set 2) and human hepatocytes experiments were reduced, alkylated, and digested on-bead using filter-aided sample preparation (Wisniewski et al., 2009) with sequencing grade modified porcine trypsin (Promega, WI). Tryptic peptides were then separated by reverse phase XSelect CSH C18 2.5 µm resin (Waters) on an in-line 150 x 0.075 mm column using an UltiMate 3000 RSLC nano system (Thermo Fisher Scientific, Lenexa, KS). Peptides were eluted using a 60 min gradient from 98:2 to 65:35 buffer A:B ratio. (Buffer A = 0.1% formic acid, 0.5% acetonitrile; buffer B = 0.1% formic acid, 99.9% acetonitrile.)

Eluted peptides were ionized by electrospray (2.4 kV) followed by mass spectrometric analysis on an Orbitrap Eclipse Tribrid mass spectrometer (Thermo Fisher Scientific, Lenexa, KS). MS data were acquired using the FTMS analyzer in profile mode at a resolution of 120,000 over a range of 375 to 1200 m/z. Following HCD activation, MS/MS data were acquired using the ion trap analyzer in centroid mode and normal mass range with a normalized collision energy of 30%. Proteins were identified by database search using MaxQuant (Max Planck Institute) with a parent ion tolerance of 3 ppm and a fragment ion tolerance of 0.5 Da. Scaffold Q+S (Proteome Software) was used to verify MS/MS based peptide and protein identifications. Protein identifications were accepted if they could be established with less than 1.0% false discovery and contained at least 2 identified peptides. Protein probabilities were assigned by the Protein Prophet algorithm (Nesvizhskii et al., 2003). Specific proteins were identified and analyzed based on unique peptide count (>2) and protein probability (>90%). Additional specific interactions of heme-human HPX with proteins, i.e. the “HPX interactome” from HepG2 cells and from primary human hepatocytes were analyzed using ontology search line analysis, e.g. PANTHER GO-Slim, with appropriate enrichment (>100->80 %) and significance that identified proteins that grouped into subsets of “biological roles” consistent with several known functions of the HPX system but also novel ones.

*Immunoblot detection of heme oxygenase 1 (HMOX1)*: To investigate the roles of TfRs and LRP1 in the endocytosis of human and rabbit HPX complexes with mesoheme or protoheme together with their regulatory effects i.e. heme-dependent heme-oxygenase 1 (HMOX1) induction was used to provide evidence of heme delivery by HPX to cells. HepG2 and primary human hepatocytes were plated in six-well plates. HepG2 cells, in exponential growth after culturing for 40 hours, were rinsed and incubated in warm, gas-equilibrated, HEPES. NaOH-buffered, serum-free media, pH 7.4 with either heme/DMSO or heme-HPX complexes in PBS for 4 h at 37^o^C. Primary human hepatocytes were incubated for 2 to 7 days and the medium replaced daily with fresh medium that had been equilibrated in the tissue culture CO2 incubator. Whole-cell extracts were prepared from PBS-rinsed cells (ice-cold) and scraped into cell lysis buffer (200 µl HEPES-buffer containing Triton-X100 lysis buffer (50mM HEPES, pH 7.4, 150mM NaCl, 0.5% Triton X-100, containing phosphatase inhibitor cocktail solutions 2 , 1X complete protease inhibitor cocktail 1, 2mM phenylmethylsulphonyl fluoride (PMSF) and used for Western analyses to detect the levels of HMOX1 protein 1:2000 (Abcam, ab-189491, Cambridge, MA) detected with secondary antibody Goat anti Rabbit IgG-HRP 1:5000 (Thermo Fisher Scientific, catalog #65-6120, Lenexa, KS) with Tubulin 1:5000 (DSHB, E7s, Iowa City, IA) as the loading control and detected with secondary antibody Goat anti Mouse IgG-HRP 1:5000 (Thermo Fisher Scientific, catalog #31430, Lenexa, KS).

# Supplementary Figures and Tables

**Supplementary Figure 1**. Characterization of surface transferrin receptor 1 isolated from HL-60 cells using mesoheme-rabbit HPX Affi-Gel 15. (A) Western blot chemiluminescence signal of proteins from Affi-Gel isolates reveals biotinylated proteins that are from the plasma membrane of surface biotinylated HL-60 cells. Sample key (lanes 1-10): non-biotinylated whole cell extracts (WCE) lanes 1 and 8, non-reduced (NR) and reduced (R), respectively. Biotinylated proteins isolated from the heme-HPX Affi-Gel lane 3 (NR) and lane 6 (R); negative controls: biotinylated proteins isolated from ovalbumin Affi-Gel, lane 2 (NR) and lane 7 (R). (B) a Coomassie stained gel with the same sample set that underwent electrophoresis simultaneously showing the location of regions excised for elution followed by LC-MS/MS analyses to identify the proteins present (Supplementary Table 1). Sample boxes labeled 1 and 3 boxes (lanes 2 and 3, respectively, are non-reduced and contain biotinylated proteins eluted from the heme-HPX Affi-Gel and the negative control ovalbumin Affi-Gel, respectively. Sample boxes labeled 6 and 7 are also from the heme-HPX Affi-Gel and the negative control ovalbumin-Affi-Gel 15, respectively, but were reduced before electrophoresis. Prestained molecular weight (Mr) standards are in lanes 5 and 9 and the approximate Mr are shown.

**Supplementary Figure 2.** Surface binding of mesoheme-rabbit HPX and evidence for its endocytosis into Rab5-positive early endosomes in HepG2 cells. (A) Co-localization of the plasma membrane marker Na^+^/K^+^ ATPaseAF546 (green color) with mHAF647RbtHPX (red color) after 1 hour incubation of HepG2 cells in medium on ice. (B) Cytofluorograms of the whole field and regions of interest (ROI) reveal positive Pearson coefficient (PC). Evidence for endocytosis of mHAF647RbtHPX within Rab5-positive early endosomes in HepG2 cells. (C) Co-localization of the early endosome marker Rab5AF488 (green color) and mHAF647RbtHPX (red color) after 10 min incubation of the HepG2 cells at 37°C. (D) Cytofluorograms of the whole field and regions of interest (ROI) reveal positive PC as shown. HepG2 scale bar = 15µm.

**Supplementary Figure 3.** Source material for this research: morphology of primary human hepatocytes after culture and medical background of the liver donors and history of the perfused human livers. (**A**). Images from an Olympus CK2 light microscope (x20) of human hepatocytes growing in 6-well plates as described in the Methods. Scale bar = 15µm (**B**). Table: summary of details of the liver donors used in these studies. Definitions: BMI body mass index; DCD donation after circulatory death; Downtime: minutes that the donor was unresponsive in the field, before life saving measures could begin; Warm time: minutes between when the heart stops and when the liver is flushed before transport. CVA cerebrovascular accident (stroke); CMV cytomegalovirus infection; HIV human immunodeficiency virus; HBV hepatitis B; HCV hepatitis C; EBV Epstein-Barr virus.

**Supplementary Table 1.** Mass Spectrometry data supporting the identification of transferrin receptor 1 (TfR1) in HL-60 Affi-Gel 15 isolates from an SDS-PAGE gel slice. Proteins whose abundance was not significantly different in eluates from the mesoheme-rabbit HPX vs. ovalbumin Affi-Gel 15 resins have been omitted. Protein identifiers are presented either as Proteins IDs (from Uniprot database), protein names, and gene names. For each protein, the number of net peptides, % coverage, and predicted molecular weight are shown. The proteins that are enriched in the mesoheme-rabbit HPX Affi-Gel eluates compared with the control ovalbumin Affi-Gel eluates are pyruvate kinase and exocyst components 1 and 3, and both, intriguingly, have links with iron and with transferrin/ TfR1 respectively, as discussed in the Results and Discussion. Log2 (HPX/control) represents the ratio of protein recovery in mesoheme-rabbit HPX/control (ovalbumin) adsorptions observed among three separate biological experiments. For each bio-experiment, protein abundances were determined as LFQ values measured in triplicate injections of each sample into the LC-MS/MS, treating technical replicates as MaxQuant fractions.

**Supplementary Table 2.** These data reveal the relative abundance of proteins from HepG2 whole cell lysates that specifically bind to holo human Tf-Affi-Gel 15 compared with the negative control, ovalbumin-Affi-Gel 15 as described in for HPX binding proteins in supplementary Table 3. The PANTHER Go Slim analyses reveal that specifically bound proteins are linked with the following biological processes include: iron ion transport, heme metabolic process, intracellular iron ion homeostasis, tricarboxylic acid cycle, transition metal ion transport, complement activation, anti-bacterial humoral immune response (see Table 2).

**Supplementary Table 3.** MS data supporting the identification of TfR1 in HepG2 whole cell extracts from mesoheme-rabbit HPX Affi-Gel 15. Proteins whose abundance was not significantly different in eluates from the HPX vs. control i.e. ovalbumin Affi-Gel 15 resins were omitted from the table. Protein identifiers are presented either as Proteins IDs (from Uniprot database) and protein names followed by the gene names. For each protein the number of net peptides identified is shown next and the % coverage of the protein amino acid sequence. Log2 (HPX/control) represents the ratio of protein recovery in mesoheme-Rabbit HPX/control (ovalbumin) adsorptions observed among three separate biological experiments. For each bio-experiment, protein abundances were determined as LFQ values measured in triplicate injections of each sample into the LC-MS/MS, treating technical replicates as MaxQuant fractions. MaxQuant LFQ protein abundances were used to calculate log2 protein ratios (mesoheme-rabbit HPX resin vs. ovalbumin resin). Significant proteins (log 2 for significance plus the extent of amino acid coverage and number of peptides) are enriched in eluates compared with the control ovalbumin are as discussed in the Results section: LRP1, Proteasome subunit β type 8, Probable tRNA pseudouridine synthase 2, Peptide D-formylase, Pro-cathepsin H, Enolase-phosphatase E1, E3 Ubiquitin-protein ligase, and Methylosome subunit plCln. These proteins are also presented as a Volcano plot in Fig. 6. See also Table 3 for the PANTHER Go Slim biological processes. (The HPX present is rabbit and is anticipated to have leached from the rabbit heme-HPX Affi-Gel resin as can occur in this isolation technique and was not included in the PANTHER analyses.)

# References

Morgan, W.T., Muster, P., Tatum, F., Kao, S.M., Alam, J., and Smith, A. (1993). Identification of the histidine residues of hemopexin that coordinate with heme-iron and of a receptor-binding region. *J. Biol. Chem.* 268(9)**,** 6256–6262. PMID:7681064

Nesvizhskii, A.I., Keller, A., Kolker, E., and Aebersold, R. (2003). A statistical model for identifying proteins by tandem mass spectrometry. *Anal Chem* 75(17)**,** 4646–4658. doi: 10.1021/ac0341261.

Paoli, M., Anderson, B.F., Baker, H.M., Morgan, W.T., Smith, A., and Baker, E.N. (1999). Crystal structure of hemopexin reveals a novel high-affinity heme site formed between two beta-propeller domains. *Nature Struct. Biol.* 6(10)**,** 926–931. doi: 10.1038/13294

Takahashi, N., Takahashi, Y., and Putnam, F.W. (1984). Structure of human hemopexin: O-glycosyl and N-glycosyl sites and unusual clustering of tryptophan residues. *Proc Natl Acad Sci U S A* 81(7)**,** 2021–2025. doi: 10.1073/pnas.81.7.2021.

Wisniewski, J.R., Zougman, A., Nagaraj, N., and Mann, M. (2009). Universal sample preparation method for proteome analysis. *Nat Methods* 6(5)**,** 359–362. doi: 10.1038/nmeth.1322.
