# Supplementary figures and images for: Tracking hemopexin intracellularly and defining hemopexin protein “interactomes” in human immune and liver cell models
Source: Front Physiol. 2025 Nov 20;16:1613917. doi: 10.3389/fphys.2025.1613917 (PMC12690290; doi:10.3389/fphys.2025.1613917)

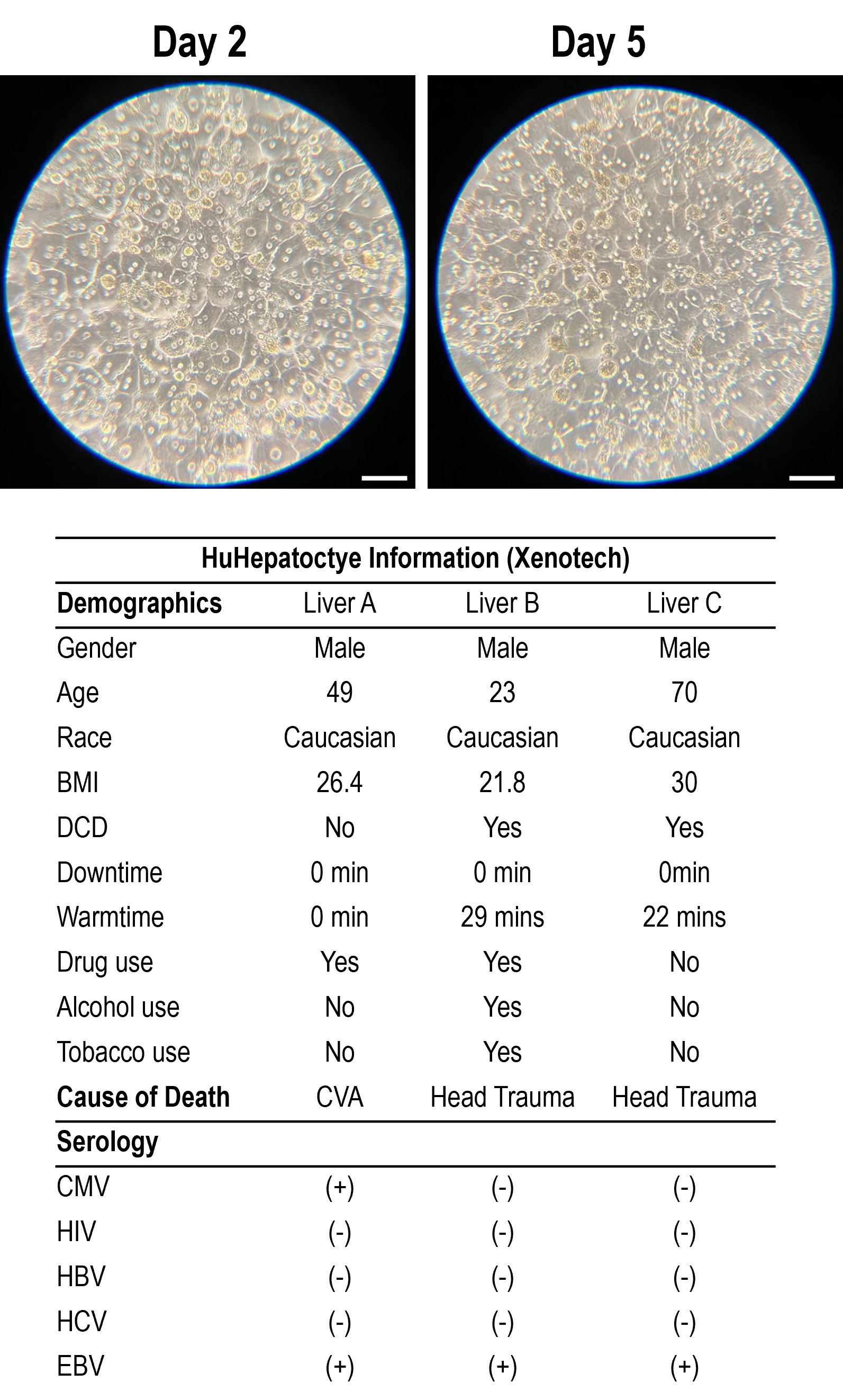

Supplement: Supplementary file 3 [file Image3.tif]

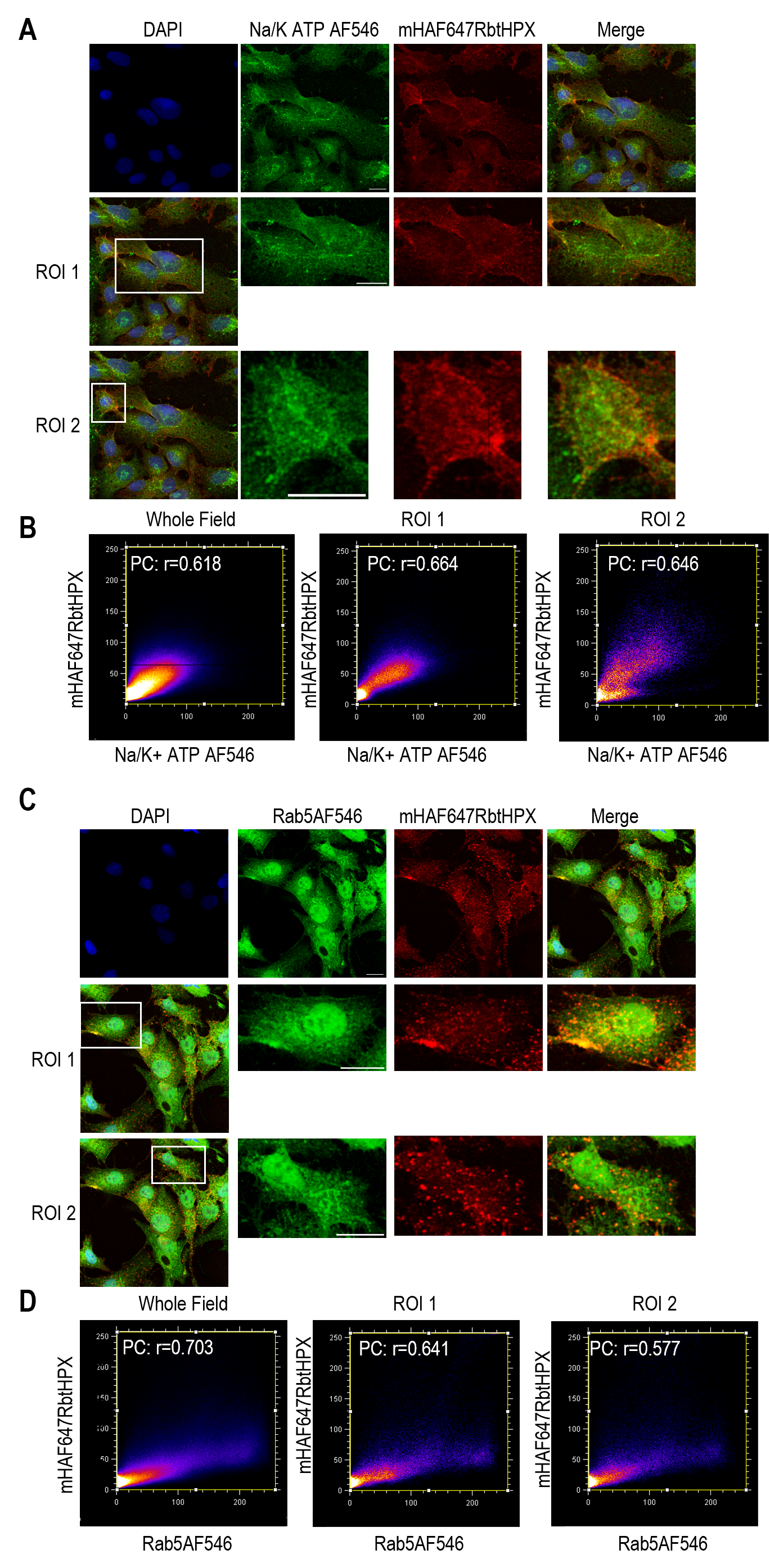

Supplement: Supplementary file 4 [file Image2.tif]

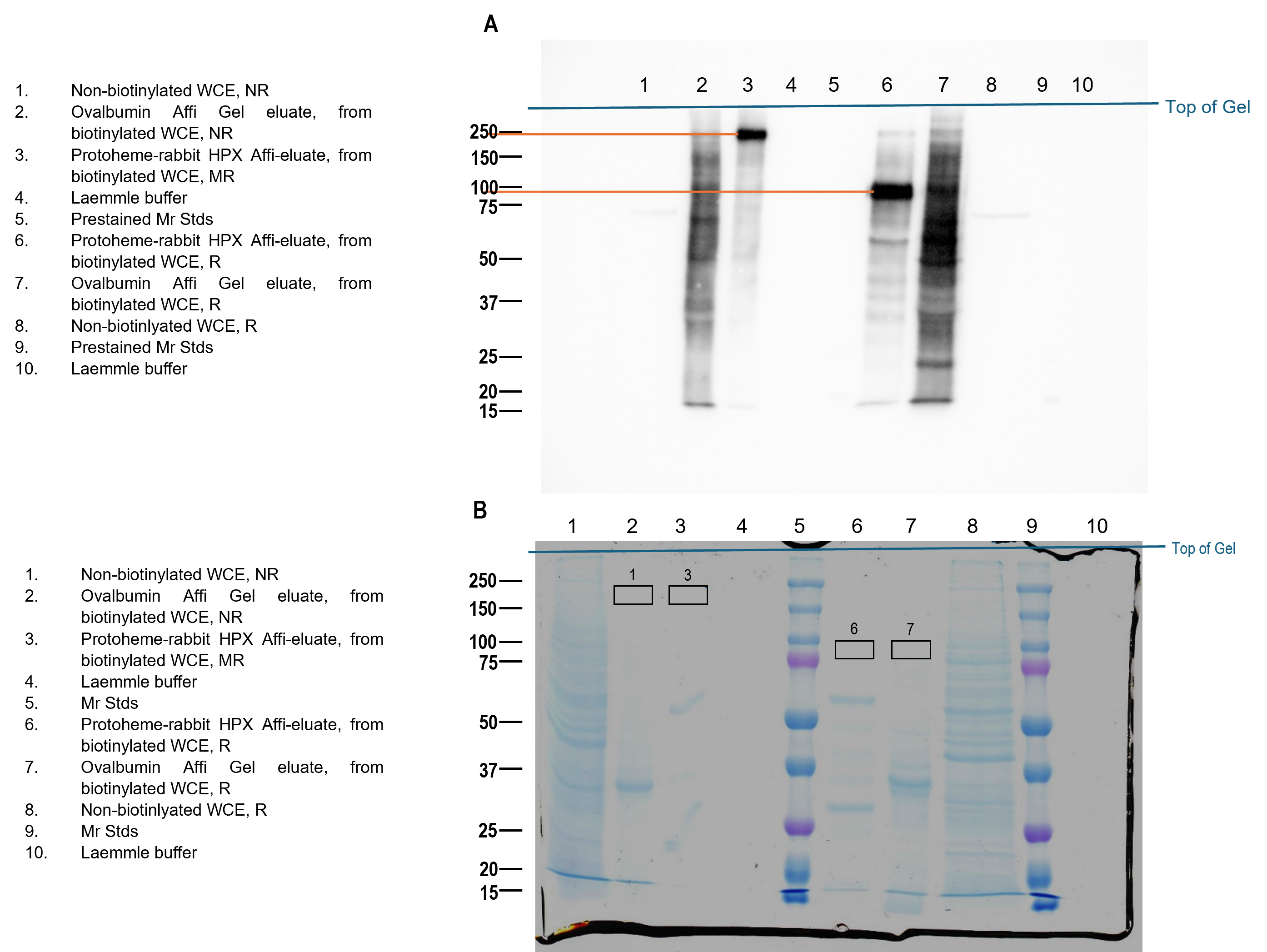

Supplement: Supplementary file 5 [file Image1.tif]
